# Clustering of Physical Activity, Diet and Sedentary Behavior among Youth from Low-, Middle-, and High-Income Countries: A Scoping Review

**DOI:** 10.3390/ijerph182010924

**Published:** 2021-10-17

**Authors:** Gabrielli Thais de Mello, Marcus Vinicius Veber Lopes, Giseli Minatto, Rafael Martins da Costa, Thiago Sousa Matias, Paulo Henrique Guerra, Valter Cordeiro Barbosa Filho, Kelly Samara Silva

**Affiliations:** 1 Research Center for Physical Activity and Health, Department of Physical Education, School of Sports, Federal University of Santa Catarina, Florianópolis 88040-900, Brazil; marcuslopes.author@gmail.com (M.V.V.L.); gminatto@gmail.com (G.M.); rafamc95@yahoo.com.br (R.M.d.C.); thiagosousamatias@gmail.com (T.S.M.); ksilvajp@gmail.com (K.S.S.); 2 Department of Medicine, Federal University of Fronteira Sul, Chapecó 89815-899, Brazil; paulo.guerra@uffs.edu.br; 3 Federal Institute of Education, Science and Technology of Ceara, Aracati 62800-000, Brazil; valtercbf@gmail.com

**Keywords:** cluster analysis, diet, exercise, sedentary behavior

## Abstract

Background: The interaction between physical activity (PA), diet, and sedentary behavior (SB) plays an important role on health-related outcomes. This scoping review (Prospero CRD42018094826) aims to identify and appraise clusters of PA, diet, and SB among youth (0–19 years) according to country income. Methods: Five databases were searched. Fifty-seven articles met the inclusion criteria. Results: Fifty-five cluster types were identified, with greater variety in high-income than lower income countries. The most prevalent profiles were “*High SB and consumption of sugar, salt, and beverages (SSB)*” (*n* = 17) and “*High PA*” (*n* = 13–5), both of which presented in all income countries. The healthiest profile, “*High PA and fruit and vegetables (F&V); Low SB and SSB*” (*n* = 12), was present in upper-middle and high-income countries, while the unhealthiest “*Low PA and F&V; High SB and SSB*” (*n* = 6) was present only in high-income countries. Conclusions: High SB and unhealthy diet (SSB) were more prevalent in clusters, mainly in high-income countries. The results support the need for multi-component actions targeting more than one behavior at the same time.

## 1. Introduction

Physical activity (PA), dietary patterns, and sedentary behavior (SB) are recognized as obesity behavioral determinants [1], which have commonly been targeted on interventions [2,3] due to their effects on energy balance. Their interaction also plays an important role in overweight [4] and other health outcomes [5,6,7] in children and adolescents. When these behaviors are individually evaluated, especially for not accounting for collinearity in traditional analyses, their effects on health outcomes can be reduced or even nullified [8]. Understanding PA, diet, and SB patterns among the pediatric population can be used to guide strategies to promote behavior change in this population [9].

A previous narrative review identified that PA, diet, and SB cluster in healthy and unhealthy patterns [10], which was also observed in recent studies [6,11]. A multicentric study conducted in ten European cities identified that 42% of adolescents were allocated to a cluster characterized by low levels of PA and SB, and high-quality diet [12]. Another study conducted in Brazil observed that 45% of 102,072 adolescents were allocated in a cluster characterized by healthy PA and diet profile, although spending almost four hours daily in SB [13]. Furthermore, these clusters have been associated with social, economic, and cultural aspects that do not affect individual behaviors equally [14,15] and may be attributed to the demographic context and population characteristics [15,16]. Socioeconomic status (SES) or its derivatives (e.g., income, education, and occupation) in a country has been recognized as an important health determinant due to its influence on people’s attitudes, experiences, and exposure to several health risk factors throughout their lives [17,18]. Thus, patterns of health-related behaviors are expected to vary between nations due to sociodemographic and cultural distinctions. For example, Collese and colleagues [15] found that European (HELENA study) and Brazilian girls (BRACAH study) have similar cluster patterns. However, among boys, a cluster characterized by higher levels of PA was observed only in the Brazilian sample. Further, Dumuid and colleagues [6] identified distinct lifestyle behavior clusters among 12 countries from low- to high-income classification. The “all-round” cluster, characterized by low screen time, healthy eating pattern, and moderate PA/SB was observed among 9 out of 12 sites, which excluded Brazil, Kenya, and South Africa. Thus, differences in PA, diet, and SB patterns in socially and economically distinct regions remain unclear.

Previous reviews have presented interesting findings on behaviors clusters among adolescents. Parker and colleagues systematically reviewed activity-related behavior typology (i.e., PA and SB), but their combination with dietary profiles were not included [19]. Another study evaluated PA, diet, and SB clusters in a non-systematic way, which limited the findings found [10]. In addition, findings on behavior profiles can be used to guide interventions in order to propose strategies to subgroups of children and adolescents to promote behavior change. Interventions with strategies aimed at individuals or subgroups are more likely to be effective in comparison to those targeted to adolescent’s population as a whole [9].

Based on previous evidence on the world’s health and income inequalities [20] and on associations between socioeconomic determinants and clusters [10,13,15], this study proposes the following advancements: (a) conducting a systematic scoping review on clusters of PA, diet, and SB among the pediatric population; (b) identifying if behavioral clusters differ according to country income; and (c) if critical appraisal within sources of evidence is found. This systematic scoping review can be used to inform readers about the state of evidence and to provide guidance for future research priorities in the clustering of obesogenic behaviors theme.

## 2. Methods

### 2.1. Protocol and Registration

This scoping review is part of a comprehensive project (PROSPERO register number: CRD42018094826) and was reported in accordance to the Preferred Reporting Items for Systematic reviews and Meta-analyses for Scoping Reviews (PRISMA-ScR, see checklist in Table S1) [21]. The search strategy included five electronic databases (PubMed, Web of Science, LILACS, Scopus, PsycINFO). The final search was conducted in December 2019 with no restriction in regard of publication year. Searches considered particularities from each database and Booleans operators and truncation symbols ($, * or ″″) were used. The final search string can be found in Table S2. Reference lists of included studies and previous reviews were examined as additional searches.

### 2.2. Eligibility Criteria

Criteria for inclusion were that the articles must: (1) include children and/or adolescents (aged 0–19 years); (2) simultaneously analyze PA, diet, and SB by applying data-based cluster statistical procedures (studies could also include additional behaviors); and (3) be published in English, Portuguese, or Spanish. Exclusion criteria was that articles must not include clinical populations (e.g., disabilities, metabolic and/or cardiovascular diseases).

### 2.3. Screening Process

Duplicates were identified and withdrew in EndNote software. Firstly, trained independent peers (GTM/RMC and GTM/MVVL) screened titles and abstracts. Discrepancies were solved by a fourth author (GM). If the relevance of an article was unclear, it was retained for full text screening. Secondly, full-text assessments were conducted (GTM/GM and RMC/MVVL) with a third reviewer (MVVL and GTM for the first and second pair, respectively) solving discrepancies. Reference list were checked by MVVL and RMC.

### 2.4. Data Extraction and Synthesis

Data were extracted by the same peers of the full-text review process. Cluster characteristics were identified by GTM and MVVL, and disagreements were also solved by consensus (GTM, MVVL, GM, and RMC).

Data extraction included: (1) general characteristics (e.g., publication year, country, design, sample size and age); (2) instruments and procedures used to measure PA, diet, and SB); PA, diet, and SB domain and components (e.g., leisure-time PA, habitual PA, fruits, vegetables, snacks, daily time spent on TV, computer, videogames), as well as other evaluated behaviors (e.g., sleep, tobacco and alcohol consumption) (see Table S3); and (3) cluster results (e.g., number of outcomes included in clustering procedures, cluster statistical approach, clusters descriptions and prevalence).

Cluster characteristics were extracted in accordance to authors’ original descriptions. When textual description was not available, quantitative data was considered. PA, diet, and SB components on each cluster were categorized as “Low”, “Average”, or “High”, and were used to define labels. For example (for a study that applied the k-means technique), a cluster characterized by screen time estimates similar to the overall sample, and by physical activity estimates higher in at least 0.30 SD above the overall sample would be classified as *High PA and Average SB*. However, as the interest is in the comparison, the “average” term was omitted from labels as commonly performed by authors when describing behavioral patterns. The cut point for classification (e.g., ±0.30 SD) varied between studies due to sample particularities and distinct clustering techniques. This is the reason we choose to label according to the authors description when properly presented. Dietary patterns, referring to ultra-processed food consumption, were named as sugar, salt, and beverages (SSB) (i.e., snacks, sweetened beverages, excessive salty foods, candies, and fried meals) and fruits, green salads, and vegetables (F&V) (i.e., fruits, vegetables, and fiber consumption). Dietary profiles that did not fit in SSB and F&V patterns were defined as “Specific Diet” (e.g., milk and meat consumption). For example, a cluster described as lower consumption of snacks and soft drinks, higher consumption of fruits and vegetables, and average time spent in PA and SB was labeled as *High F&V and Low SSB*. The “Average” category was omitted from labels.

Self-reported instruments applied to measure PA, diet, and SB were classified as: (1) Defined, if referred to consolidated or previously validated instrument; (2) Undefined, if authors did not clearly report question and/or response options, as well as the reference of the instrument used; (3) Undefined–Reproductible, if authors clearly reported question and response options allowing for replication but did not mention the reference used.

A country’s income classification was performed according to The World Bank (low income, lower middle income, upper middle income and high income) considering data collected year of each study (https://datahelpdesk.worldbank.org/; accessed at 7 July 2021).

A narrative synthesis of findings was conducted and structured around the descriptive characteristics of included studies (e.g., year of publication, continent, sample procedures, instruments, and others). Additionally, behaviors (PA, diet, and SB) were described considering: details of their components; measurement instrument; and number of outcomes used in clusters procedures. In addition, we detailed the data-based cluster statistical procedures used to identify number and clusters types found in the studies. The descriptive analysis was based on the total number of studies; thus, articles originated from the same study were represented by the article with the largest sample. Thus, in cluster description results, the same clusters from the same population presented in different articles were reported once. Since this, cluster descriptions were made according to analysis used to identify patterns: (a) cluster analysis (i.e., k-means, Ward’s method, latent class analysis, and latent profile analysis) and (b) dimensionality reduction procedures (i.e., principal component analysis, multiple corresponding analysis, and factorial analysis).

### 2.5. Critical Appraisal of Individual Sources of Evidence

We performed a critical appraisal of included studies to map the quality research on clustering of obesogenic behaviors in different countries as an optional step for scoping reviews and a fundamental element for the research implications of this study. For this, an adapted 17-point version of the quality assessment tool for quantitative studies of the Effective Public Health Practice Project (EPHPP) was used [22]. Original papers were assessed by four methodological domains: (1) selection bias (sample characteristics in relation to the review target population (*strong* or 1: ≥80%; *moderate* or 0: 79–60%; *weak* or −1: ≤60%)); (2) study design (information about study representativeness (yes = 1; no = 0); described sampling methods (yes = 1; no = 0); appropriate sampling method (random = 1; not described = 0; convenience = −1))—*strong* for 1 in all three items, *moderate* for combinations: 1-1-0, 1-0-1, 1-0-0, and 0-0-1, and *weak* for all other combinations; (3) information about instruments to evaluate PA, diet, and SB (report of its previous validation (yes = 1; no = 0), and information that would enable reproducing PA, diet, and SB assessment (yes = 1; no = 0))—studies using an accelerometer to measure PA and/or SB were assigned a score of "1", that is, it was considered that there was a previous validation report of the instrument—*strong* for 1 in both outcome items, and *weak* for all other combinations; and (4) flow of people throughout the study (report in terms of numbers and/or reasons (yes = 1; no = 0) and percentage of participants completing the study (≥80% = 1 or strong; 60–79% = 0 or moderate; ≤59% = −1 or weak))—*strong* for 1 in both items or 0 and 1, *moderate* for combinations 1 and 0 or 0 and 0, and *weak* for all other combinations. The classification (low (strong), moderate (moderate) and high (weak)) for each domain was performed based on a study distribution (see Table S3). Two independent reviewers (GTM and GM) assessed the risk of bias in included studies, and a third reviewer evaluated disagreements (MVVL).

## 3. Results

### 3.1. Selection of Sources of Evidence

**Figure 1:**
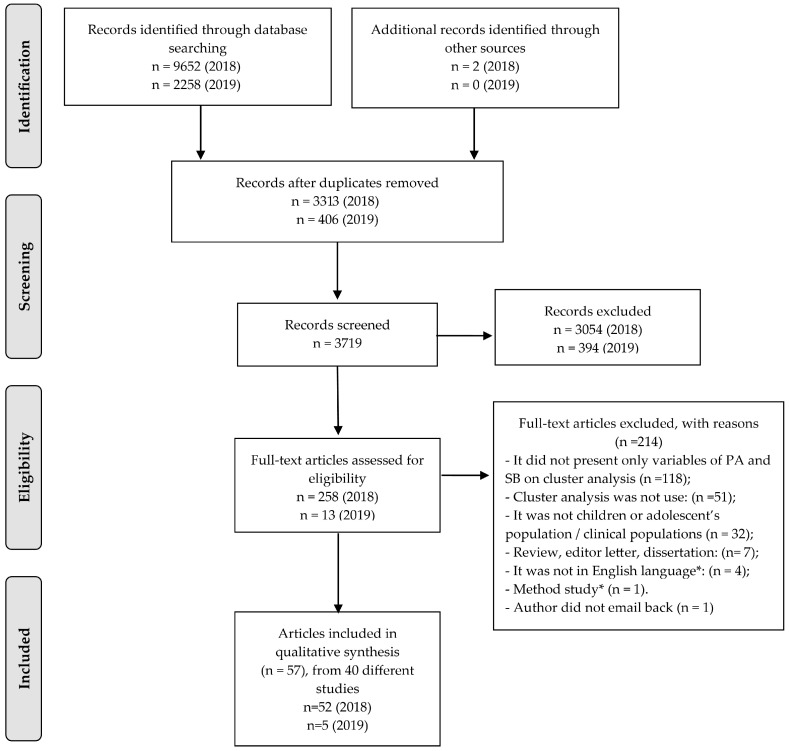
Flowchart of studies selection procedure in accordance to PRISMA flow diagram. * French (*n* = 1), German (*n* = 1), and Polish languages (*n* = 2). * Explained how to use cluster analysis—did not present original findings. PA: physical activity.

A total of 11,910 articles were identified, of which 57 were included in the present work. Of these, 40 different studies were identified. A summary of each review phase and reasons for exclusion is available in the flowchart of Figure 1.

### 3.2. Characteristics of Sources of Evidence

**Figure 2:**
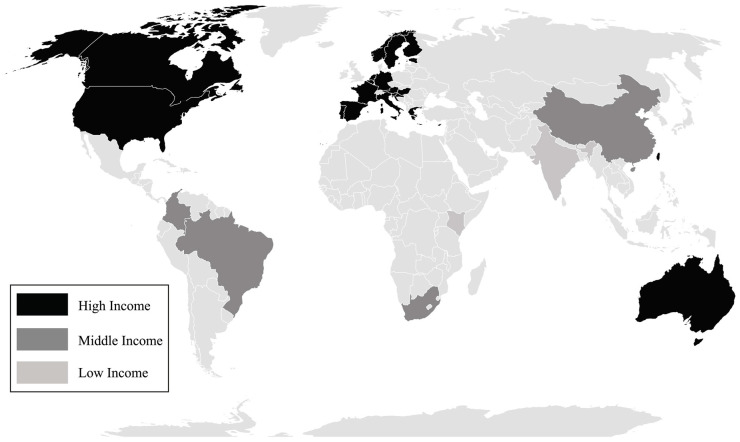
Countries included in the scoping review according to income level. Source: by the authors.

Characteristics of studies are present in Figure 2 and Table S4. Studies from the same sample data were presented once, considering the largest sample (Table S6). Three articles used HBSC data with samples from their respective countries (Italy [23], Finland [24], and Portugal [25]). Thus, we considered three articles to represent the HBSC study. Forty-two studies were considered to describe the characteristics of the studies. The publication year ranged from 2006 [26,27] to 2019 [28], and the majority included cross-sectional design (*n* = 26) [6,23,24,25,26,29,30,31,32,33,34,35,36,37,38,39,40,41,42,43,44,45,46,47,48,49]. The studies were developed in 29 different countries, the majority were carried out in USA (*n* = 6), Brazil (*n* = 6), and Australia (*n* = 4), and five [6,11,34,35,50] provided data from more than one country. Regarding country income, 35 studies [11,23,24,25,26,27,29,30,32,33,34,35,36,37,38,40,43,44,45,47,48,49,50,51,52,53,54,55,56,57,58,59,60,61] were developed in high-income countries, followed by six [31,39,41,42,46,62] in upper middle-income countries, and one [6] involved data on countries with more than one income.

The age group ranged from two [50,63] to nineteen [13,42] years. Most studies exclusively investigated adolescents (*n* = 23) [23,24,25,26,28,31,32,34,35,37,39,40,41,42,45,46,47,48,55,57,59,60,62], nine [6,29,30,33,38,43,49,51,54] both children and adolescents, and seven (*n* = 7) [11,44,50,52,53,58,61] only children. In three studies [27,36,56], the sample was composed of children/adolescents but did not report the age group. The sample size ranged from 284 [7] to 109,104 [39] participants, representing a total of 362.471 children and adolescents.

### 3.3. Critical Appraisal within Sources of Evidence

Disagreement percentage among risk of bias evaluators was approximately 30.7% (kappa = −0.03–1.0), ranging from 5.2% (“Question 6. Is there information that enables replicating the tool?” for diet) to 62.1% (“Question 8. Indicate the percentage of participants completing the study”).

**Figure 3:**
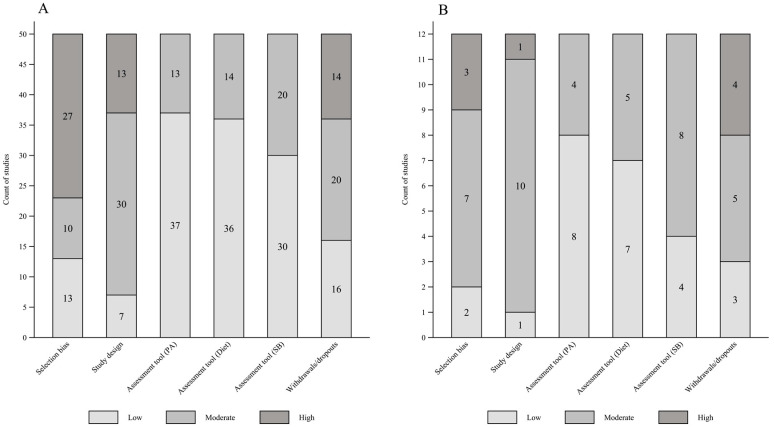
Risk of bias assessment of studies from high- (**A**), and middle-income (**B**) countries.

In risk of bias assessment (see Table S4), several studies from high-income countries failed to achieve at least 60% of the eligible response, which compromised the sample representativeness. This occurred at a lower frequency among studies from middle-income countries. In addition, a percentage of ≥80% of participants who completed the study was observed in less than half of included studies, regardless of the income level of the countries. On the other hand, almost all studies in all income levels, except one [32], presented information that enables replication of the tool of PA, diet, and SB. In Figure 3, a higher frequency of studies with a high risk of bias was observed for items selection bias among those from high-income countries and assessment tool of SB for studies from middle-income countries. The assessment tool of PA and diet were the items most frequently classified with low risk of bias among studies for both income levels of the countries (Figure 3). In the two studies from low-income countries, a low risk of bias for the assessment tool of PA and diet was observed. Half the studies showed a low risk of bias, and half a moderate risk for the selection bias, assessment of SB, and withdrawals/dropout items. For the study design item, one study was classified as having a moderate risk of bias and the other study with a high risk of bias (data not shown).

### 3.4. Behavior Measurement

Information about assessment tool classifications is available in Figure S1. Objective measures were identified on five [6,11,33,51,60] and two [51,60] studies to evaluate PA and SB, respectively. Questionnaires were the most prevalent instrument used to measure PA (*n* = 35) [5,11,15,24,26,27,28,29,30,31,32,34,35,36,37,38,39,40,41,42,43,44,45,46,47,48,49,50,52,53,54,55,56,57,59,61,62], diet (*n* = 33) [5,7,11,15,24,26,27,28,31,32,33,35,36,37,39,40,41,42,44,45,46,47,48,49,50,51,52,53,54,55,56,57,59,61,62], and SB (*n* = 37) [5,7,11,15,24,26,27,28,29,30,31,32,33,34,35,36,37,38,39,40,41,42,43,44,45,46,47,48,49,50,52,53,54,55,56,57,59,61,62]. Most questionnaires applied [5,6,11,15,26,27,32,34,35,37,38,39,40,41,42,43,45,46,47,48,49,50,51,52,56,57,59,61,62] were consolidated or previously validated to PA (*n* = 85; 77.6%), diet (*n* = 83; 9.2), and SB (*n* = 93; 49.5%). However, six [29,30,46,53,55,57], four [26,33,53,55], and twelve [6,29,30,33,41,42,45,46,47,53,55,57] studies that used undefined questionnaires (authors did not clearly report question and/or response options, and instrument reference) for PA, diet, and SB, respectively. One [58] study used a diary to evaluate PA, diet, and SB; six studies [29,30,34,38,43,60] evaluated diet applying recalls.

All outcomes for PA, diet, and SB used in cluster procedures can be observed in Figure S2). The most common outcomes for PA were *weekly* PA (*n* = 22 articles) [12,23,25,31,32,36,37,39,40,41,43,46,48,52,54,55,57,59,61,62,64,65], followed by *weekly leisure-time* PA (*n* = 15 articles) [5,13,24,26,27,28,38,39,45,47,50,55,56,61,66] and *accelerometer measured* PA (*n* = 9 articles) [4,6,7,33,51,60,63,67,68]. *Daily* PA, PA in *physical education classes*, and *daily leisure-time* PA were used by seven [15,34,35,49,53,58,69], six [11,39,42,44,55,59], and four [29,30,70,71] articles, respectively. Only one [44] article used *leisure-time* PA (i.e., yes or no).

For SB, *daily* screen time was the most commonly used outcome (*n* = 30 articles) [6,7,15,23,28,29,30,33,34,35,36,37,38,40,41,43,46,47,53,56,57,58,61,66,67,68,69,70,71] followed by *daily* TV time (*n* = 16 articles) [4,5,13,23,24,37,39,42,44,49,50,51,54,55,58,65]. Other articles used *daily* videogame time (*n* = 9) [5,13,23,24,37,49,54,55,65], *daily* computer time (*n* = 8) [23,24,37,49,54,55,58,65], *weekly* TV time (*n* = 7) [27,32,48,52,59,63,64], and *weekly* computer time (*n* = 6) [27,32,48,52,59,64]. *Daily* non-screen activities [5,13,34,39], *daily* stationary time [4,6,51,60], and *weekly* screen time [25,45,50,62] were used in four articles. Finally, three [45,48,59] articles used *weekly* non-screen activities, three [12,31,49] articles used *daily* SB, two [32,59] articles used *weekly* videogame time, and only one [26] article used *weekly* SB.

Regarding diet, the outcomes most used were *daily* consumption of F&V (*n* = 23) [4,11,15,24,26,30,33,36,38,42,44,48,49,51,53,54,58,59,61,65,69,70,71], followed by *weekly* consumption of SSB (*n* = 21) [5,13,23,25,31,37,39,40,41,44,50,52,54,55,56,62,63,64,65,67,68], *weekly* consumption of F&V (*n* = 20) [5,13,23,25,27,31,37,39,40,41,50,52,55,56,62,63,64,67,68], *daily* consumption of SSB (*n* = 17) [4,11,15,26,30,35,38,42,49,51,53,58,59,61,69,70,71], *weekly* consumption of fast foods (*n* = 14) [5,13,23,27,31,37,39,40,52,54,55,61,64,65], and diet score (*n* = 12) [6,7,12,29,43,45,46,47,57,60,66,67]. Other articles used *daily* consumption of diverse foods (e.g., dairy, grain, beans, and/or fiber) (*n* = 8) [24,30,38,44,49,59,70,71], *daily* consumption of fast foods (*n* = 6) [24,38,49,53,58,59], *daily* consumption of meats (e.g., bovine, chicken, fish, and/or pork) (*n* = 6) [30,38,49,59,70,71], *weekly* consumption of diverse foods (*n* = 5) [39,52,55,64,67], *weekly* consumption of meats (*n* = 3) [52,61,64], and *monthly* consumption of SSB (*n* = 2) [28,33]. *Monthly* consumption of fast foods [28], *monthly* consumption of F&V [28], and *monthly* consumption of diverse foods [33] were used once in each article. Additionally, one study evaluated dietary balance, dietary diversity, dietary quality, and meal index [34].

### 3.5. Analytical Approaches

Several data-driven clustering methods were used to determine clusters (see Figure S3). From 57 articles, 26 used k-means cluster analysis [4,6,7,11,12,15,23,24,25,28,34,35,39,41,42,43,45,47,50,51,56,59,62,63,66,67], and 15 of these applied the combination of Ward and k-means methods to identify the number of meaningful clusters to assign individuals into clusters [6,7,11,15,34,35,41,42,43,47,50,62,63,66,67]. Only one study exclusively applied the Ward method [33]. The use of latent class analysis was observed from 2011 and increased in 2017 [36,37,53,54,55,58,60,65,68,69]. A similar trend was observed for the use of the two-step cluster analysis [5,13,32,40,48,49,57].

### 3.6. Cluster Profile

A total of 55 cluster types were identified. A large number of studies used four [5,13,33,34,35,40,42,43,50,56,62,63] outcomes in data-driven procedures. In addition, outcomes number ranged from three [12,47,60,66] to 41 [61] (see Figure S4). Twenty-five studies identified clusters considering only three behaviors (PA, diet, and SB) [4,5,6,7,12,13,25,26,29,33,34,37,38,42,45,47,50,51,52,56,60,62,63,64,66]. Studies included other behaviors in clustering procedures beside these three, such as: sleep (*n* = 13) [11,15,24,30,35,41,43,49,58,67,68,70,71], risk behaviors (*n* = 11) (e.g., aggression, alcohol, tobacco, drugs, unprotect sex, bullying, violence) [23,28,31,32,36,46,48,54,59,61,65], weight control behavior (e.g., vomiting or taking laxatives or pills)(*n* = 4) [36,53,55,69], weight perception (*n* = 1) [69], PA environment (*n* = 1) [59], family-related variables (e.g., family structure and medical history, father and mother PA levels, and excess weight) (*n* = 2) [44,61], socioeconomic and demographic aspects (e.g., schooling, birth data) (*n* = 1) [61], hygiene (*n* = 1) [49], and diet habits (e.g., eating with parents/guardians, eating in front of television or studying and having breakfast) (*n* = 1) [39]. Nineteen studies stratified clusters by sex [12,15,24,28,31,35,36,39,41,42,43,45,49,50,59,60,62,63,67], five by age [4,38,43,51,63], and one by country [6].

### 3.7. Cluster Analysis

**Figure 4:**
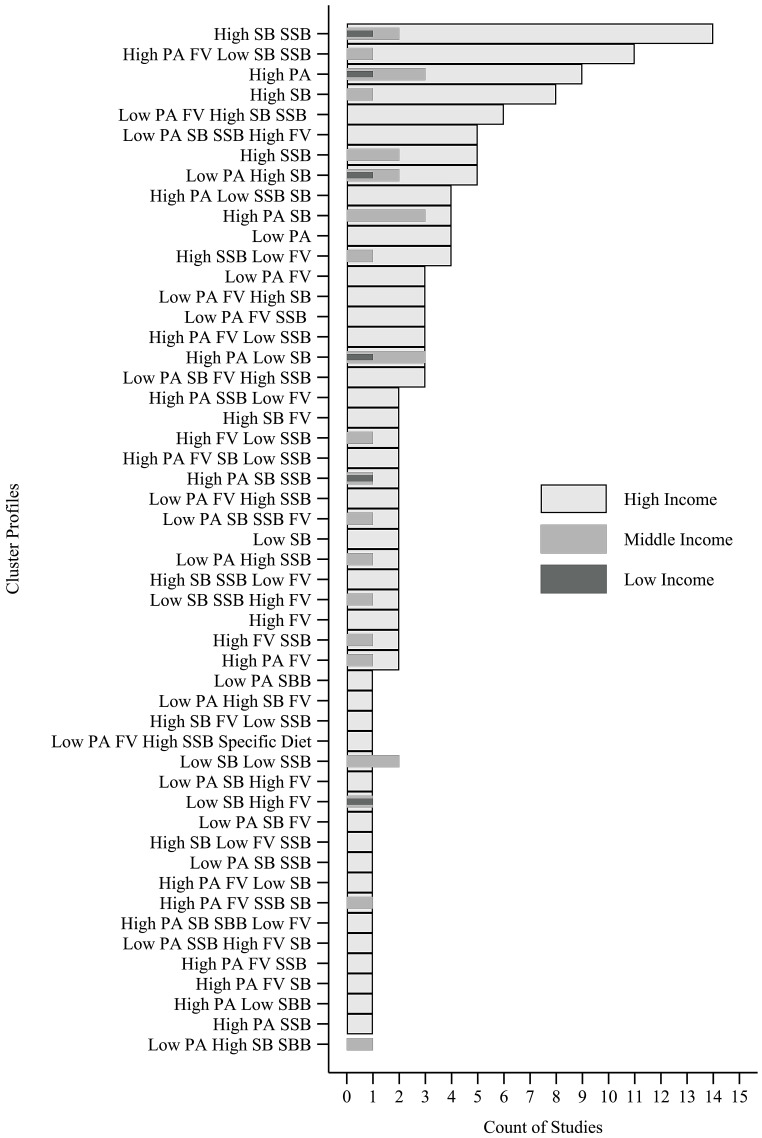
Characteristics of clustering patterns of obesogenic behaviors applying cluster analysis (latent class analysis, latent profile analysis, two-step and K-means) across studies. Middle income includes lower-middle- and upper-middle-income countries. F&V: fruits and vegetables; SSB: sugar-sweetened beverages; SB: sedentary behavior; PA: physical activity. Country income classified by The World Bank (https://datahelpdesk.worldbank.org/, accessed at 7 July 2021) according to year of data collected of each study.

By applying cluster analysis (i.e., k-means, Ward’s method, latent class analysis, and latent profile analysis), 51 cluster types were identified, and 42 included at least one negative behavior (e.g.; low consumption of F&V). Two [11,67] studies identified clusters considering a sample of more than one country income levels and were not included in counts. Clusters that appeared the most across studies presented in all income classifications, Figure 4, were the “*High SB and SSB*” (*n* = 17) [4,6,7,15,24,33,35,37,49,51,53,55,57,63,68,69], “*High PA*” (*n* = 13) [7,33,36,41,42,47,50,57,59,62,63,66], “*Low PA High SB*” (*n* = 8) [4,6,7,42,51,54,58,62,65], and “*High PA and Low SB*” (*n* = 7) [6,7,15,41,42,48]. Cluster type “*High SB*” (*n* = 9) [12,23,32,35,36,41,50,59,62,63] was found only in upper middle and high-income countries. The healthiest, characterized by all behaviors being healthy, “*High PA and F&V Low SB and SSB*” profile (*n* = 12) [11,15,24,25,28,33,34,37,41,43,55,56,58,60], was present only in upper-middle- and high-income countries, while the unhealthiest, characterized by all behaviors being unhealthy, “*Low PA and F&V High SB and SSB*” profile (*n* = 6) [11,24,28,45,47,56,60,66] was present only in high-income countries.

### 3.8. Dimensionality Reduction Techniques

**Figure 5:**
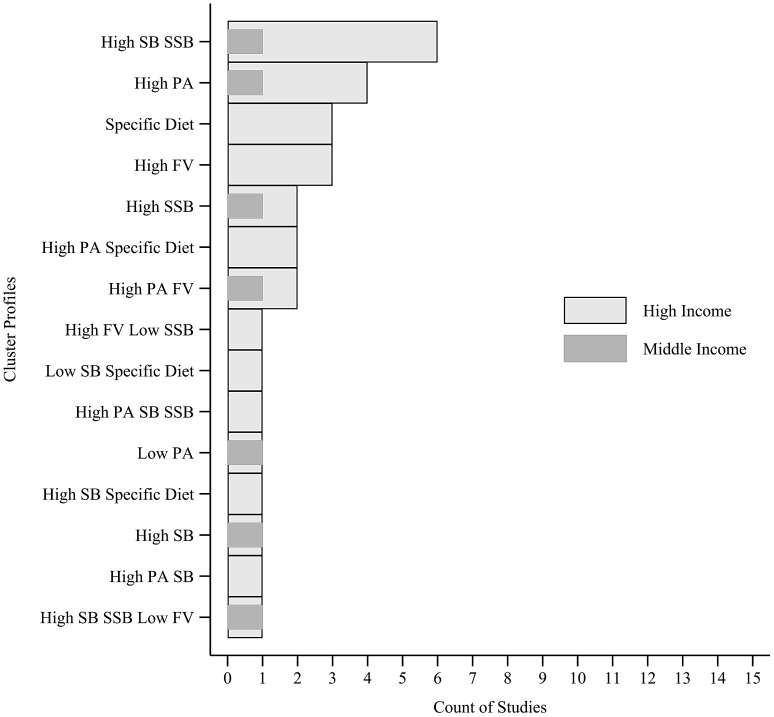
Characteristics of clustering patterns of obesogenic behaviors applying factors procedures (principal component analysis, factorial analysis and multiple corresponding analysis) across studies. F&V: fruits and vegetables; SSB: sugar-sweetened beverages; SB: sedentary behavior; PA: physical activity. Country income classified by The World Bank (https://datahelpdesk.worldbank.org/, accessed at 7 July 2021) according to year of data collected of each study.

By applying dimensionality reduction procedures (i.e., principal component analysis, multiple corresponding analysis, and factorial analysis), 15 cluster types were identified, and nine included at least one negative behavior (Figure 5). The two most prevalent cluster types found in high-income countries were also present in upper-middle-income countries. There was no evidence from low-income countries, and few cluster types were found in upper middle-income compared to high-income countries. A large proportion of studies reported clusters characterized by “*High SB and SSB*” (*n* = 7) [26,27,30,31,52,64,70,71], followed by “*High PA*” (*n* = 5) [29,30,61,64,70,71], “*Specific Diet*” (*n* = 3) [30,44,70,71], and “*High F&V*” consumption (*n* = 3) [30,52,70,71].

## 4. Discussion

This scoping review found that sundries data-driven procedures and diverse PA, diet, and SB outcomes have been used to identify clusters behaviors. The present results identified 55 cluster types in children and adolescents, and a high diversity of their types was found in data-driven cluster analysis procedures. Studies from low- and upper-middle-income countries were less well represented than those from high-income countries. The types clusters identified presented co-occurrence of healthy and unhealthy behaviors; however, unhealthy clusters were more prevalent.

### 4.1. Risk of Bias

Independently of country income, the risk of bias for sample selection is high/moderate for most of the studies. Contrarily, for design, withdrawals, and dropouts, the risk of bias was low for most studies. This result indicates that the studies’ representativeness of their target population, as well as the losses and withdrawal rate and participants who completed the study, has not been reached or is poorly reported among studies. In addition, knowing the withdrawals and losses of a study, as well as its reasons, enables a better interpretation of results. In this sense, cluster studies could report the selection process of participants, losses, and withdrawals more comprehensively. SB measurement was the third item with the highest frequency of high risk of bias. The lack of standardized instruments to measure SB makes comparison among studies difficult.

### 4.2. Studies Characteristics

Studies regarding the clustering of PA, diet, and SB are relatively recent, as the oldest publication included in this review was conducted in France and Taiwan in 2006. In addition, Europe was the continent with the largest number of included studies. This result may indicate the intensification of debate in high-income countries about this issue. Once sociodemographic outcomes seem to affect cluster formation [10], investigating obesogenic clusters in low- and middle-income countries is also necessary to improve the understanding on the topic. In addition, most studies investigated only adolescents and more studies investigating children are necessary. Once these unhealthy behaviors start at the beginning of childhood, remaining in adolescence and frequently in adulthood [72].

Questionnaires were the instrument most commonly used, and some studies [6,7,23,26,29,30,33,46,53,55,57,66,69] did not report sufficient information to replicate the instrument measurement for PA [23,29,30,46,53,55,57,69], SB [6,7,23,29,30,33,46,53,55,57,66,69], and diet [23,26,33,53,55,69]. Objective measures were used by few studies [4,6,7,33,37,51,60,63,67,68], being restricted to PA and SB assessment. Recalls and diaries to evaluate diet behavior were also less frequently observed than in questionnaires [29,30,34,38,43,58,60]. The lack of information on the instruments used is not the factor that determines the formation of clusters; however, the lack of validated and replicable instruments makes comparisons among studies difficult.

### 4.3. Outcomes

Different outcomes for PA, diet, and SB were analyzed. The number of outcomes observed in PA was smaller compared to diet and SB. Weekly PA and daily screen time were the most commonly used PA and SB outcomes, respectively. The dietary outcomes used in studies varied according to consumption frequency, such as daily or weekly consumption of F&V, SSB, meat, and diverse foods (e.g., milk). Thus, in contrast to diet variety outcomes (treatment variables) simultaneously presented in cluster procedures (e.g., consumption of fruits, ultra-processed foods, milk, and meat), only few studies analyzed more than one of PA and SB outcome simultaneously. PA, diet, and SB are complex behaviors characterized by multiple components that need to be available. Therefore, future studies should explore other outcomes of these behaviors, such as volume and different types of PA and screen time components such as cellphone time, which differently affect health.

### 4.4. Analysis

There was substantial heterogeneity in the types of clustering methods used, varying from factor-based approaches (e.g., exploratory factorial analysis) to cluster analysis (e.g., k-means and latent class analysis). If the aim is to identify cluster behaviors, both types of methods seems to be efficient, which is similar to findings reported in previous study [8]. It is noteworthy that cluster analysis has only recently been applied. It seems that over time, the authors had used cluster methods that minimize the arbitrariness in clustering formation and started to use criteria to establish the number of clusters (models fit); however, the subjectively is reduced and/or conditioned according to advance in analyses. In addition, the subjectivity in cluster labels was considerable present, and many times, cluster was named and characterized according to the “main behaviors” (the ones which present extreme values in the cluster). It is important to consider that labeling is a matter of transforming data into text that is more intelligible. However, authors should include a very comprehensive description of each variable for each cluster. When analysis allows, it is important to report the prediction importance of each variable to form the cluster (e.g. PA could discriminate population more than diet).

### 4.5. Clusters

Diverse cluster types were found, and the two most prevalent were present in all country income levels and stand out in terms of characteristics. The most prevalent clusters in decreasing order were characterized as “*High SB and SSB*”, “*High PA*”, and “*High PA and F&V Low SB and SSB*”. From the 55 cluster types, 43 profiles included at least one negative behavior in distinct combinations. The most common cluster had a combination of high time in SB with high consumption of SSB foods. A possible explanation for this finding is that watching television makes individuals eat more because they are distracted, which reduces internal satiety due to the delay of normal mealtime satiety [73,74,75,76]. Another explanation is the high number of advertisements that screen users are exposed to, which may influence the type of food consumed [77]. In addition, watching television is associated with poorer diet quality, including high consumption of SSB foods [73,78].

The two other most prevalent clusters types were “*High PA*” cluster, present in all country income levels, and “*High PA and F&V Low SB and SSB*”, present only in upper-middle- and high-income countries. These cluster types results corroborate with other studies, which emphasize that PA is positively associated with healthier eating habits and better quality of diet [26,79] and negatively associated with consumption of unhealthy foods [26,80,81]. However, no studies were found in the literature comparing clusters behaviors with country income levels. The unhealthiest cluster type (*Low PA and F&V High SB and SSB*) was present only in high-income countries. Even so, it is worth highlighting that more than 75% of cluster types had the presence of at least one unhealthy behavior. This predominance of unhealthy clusters in children and adolescents supports the need for the development of multi-component actions targeting more than one behavior at the same time.

### 4.6. Strengths and Limitations

To the best of our knowledge, this was the first study to systematically review clusters of PA, diet, and SB in children and adolescents. Another positive point is that this study showed cluster types of these behaviors by countries of different incomes. One of the limitations of this study was the subjectivity of cluster data extraction; however, a sequence of criteria and agreement was used, so that parsimonious information was obtained. Since this, the wide range of instruments used to measure PA, diet, and SB as well as variation of outcomes within each behavior may have interfered to more intelligible/readable synthesis of the present results. In addition, some articles included behaviors other than PA, diet, and SB, and the comparability with studies that did not include these are complex. It is noteworthy that strong differences and/or similarities between cluster type and country income categories may not be found due to the low number of studies carried out in lower income strata. All these aspects should be considered when interpreting the results.

### 4.7. Futures Researches

Our study identifies the number and cluster types according to country income. However, we could not conclude that clusters in low- and middle-income countries are equivalent to those of high-income countries, as: (I) there are few studies using data-driven cluster procedures in countries with lower incomes, mainly in low-income countries; (II) there is high bias in the sample selections; (III) a high variety of instruments and indicators were used for each behavior; and (IV) there is a lack of information about validity of the instruments used. Future studies should be developed in countries with lower incomes. In addition, they should improve methodological aspects, including more reliable measurements and representative samples. In addition, investigations should identify how cluster behaviors vary over time, and the effect of interventions considering cluster behaviors. No papers included in the review used longitudinal data driven cluster procedures.

## 5. Conclusions

Types of clusters considering PA, diet, and SB were identified, and even the low number of studies developed in lower income countries allowed differences in obesogenic behaviors patterns to be identified. Research on this theme has gained scientific interest in recent years; however, methodological fragilities in the studies were identified, especially in the sample selection and the quality of instruments. High SB and unhealthy diet (SSB) were more prevalent in clusters, mainly in high-income countries. The results support the need for multi-component actions targeting more than one behavior at the same time.

## Data Availability

Not applicable.

## Supplementary Materials

The following are available online at https://www.mdpi.com/article/10.3390/ijerph182010924/s1, Table S1: PRISMA-ScR, Table S2: Search of all strategy checklist, Table S3: Behaviors included in clustering procedures along with physical activity, diet, and sedentary behavior; Table S4: Methodological Characteristics of included studies, Table S5: Assessment of the bias risk of studies, Table S6: Articles from their respective studies, Figure S1: Instruments used and questionnaires classification according to each behavior, Figure S2: Behavioral outcomes used to define PA, diet, and sedentary behavior in clustering procedures, Figure S3: Use of statistical procedures to evaluate the clustering between physical activity, diet and sedentary behavior among children and adolescents, Figure S4: Quantity of outcomes used in clusters procedures.
