# Supplementary material for: Clustering of Physical Activity, Diet and Sedentary Behavior among Youth from Low-, Middle-, and High-Income Countries: A Scoping Review
Source: Int J Environ Res Public Health. 2021 Oct 17;18(20):10924. doi: 10.3390/ijerph182010924 (PMC8535526; doi:10.3390/ijerph182010924)
Supplement: Supplementary file 1 [file ijerph-18-10924-s001.zip › ijerph-1379554-SI.pdf]

Research on the clustering behaviors among young people from low-, middle- and high-income countries: a scoping review

Table S1. PRISMA-ScR checklist

Preferred Reporting Items for Systematic reviews and Meta-Analyses extension for Scoping Reviews (PRISMA-ScR) Checklist

SECTION	ITEM	PRISMA-ScR CHECKLIST ITEM	REPORTED ON PAGE #
**TITLE**			
Title	1	Identify the report as a scoping review.	Page 1. Title.
**ABSTRACT**			
Structured summary	2	Provide a structured summary that includes (as applicable): background, objectives, eligibility criteria, sources of evidence, charting methods, results, and conclusions that relate to the review questions and objectives.	Page 1
**INTRODUCTION**			
Rationale	3	Describe the rationale for the review in the context of what is already known. Explain why the review questions/objectives lend themselves to a scoping review approach.	Page 3
Objectives	4	Provide an explicit statement of the questions and objectives being addressed with reference to their key elements (e.g., population or participants, concepts, and context) or other relevant key elements used to conceptualize the review questions and/or objectives.	Page 4
**METHODS**			
Protocol and registration	5	Indicate whether a review protocol exists; state if and where it can be accessed (e.g., a Web address); and if available, provide registration information, including the registration number.	Page 4. Topic Protocol.
Eligibility criteria	6	Specify characteristics of the sources of evidence used as eligibility criteria (e.g., years considered, language, and publication status), and provide a rationale.	Page 4. Topic Eligibility Criteria.
Information sources*	7	Describe all information sources in the search (e.g., databases with dates of coverage and contact with authors to identify additional sources), as well as the date the most recent search was executed.	Page 4. Topic Protocol.
Search	8	Present the full electronic search strategy for at least 1 database, including any limits used, such that it could be repeated.	Page 5. Supplementary material Table S2.

SECTION	ITEM	PRISMA-ScR CHECKLIST ITEM	REPORTED ON PAGE #
Selection of sources of evidence†	9	State the process for selecting sources of evidence (i.e., screening and eligibility) included in the scoping review.	Page 6. Topic Data extraction and Synthesis.
Data charting process‡	10	Describe the methods of charting data from the included sources of evidence (e.g., calibrated forms or forms that have been tested by the team before their use, and whether data charting was done independently or in duplicate) and any processes for obtaining and confirming data from investigators.	Page 6. Topic Data extraction and Synthesis.
Data items	11	List and define all variables for which data were sought and any assumptions and simplifications made.	Page 6. Topic Data extraction and Synthesis.
Critical appraisal of individual sources of evidence§	12	If done, provide a rationale for conducting a critical appraisal of included sources of evidence; describe the methods used and how this information was used in any data synthesis (if appropriate).	Page 7. Topic Critical appraisal of individual sources of evidence.
Synthesis of results	13	Describe the methods of handling and summarizing the data that were charted.	Page 6. Topic Data extraction and Synthesis.
**RESULTS**			
Selection of sources of evidence	14	Give numbers of sources of evidence screened, assessed for eligibility, and included in the review, with reasons for exclusions at each stage, ideally using a flow diagram.	Page 9
Characteristics of sources of evidence	15	For each source of evidence, present characteristics for which data were charted and provide the citations.	Page 9
Critical appraisal within sources of evidence	16	If done, present data on critical appraisal of included sources of evidence (see item 12).	Page 10
Results of individual sources of evidence	17	For each included source of evidence, present the relevant data that were charted that relate to the review questions and objectives.	Page 6 to 14
Synthesis of results	18	Summarize and/or present the charting results as they relate to the review questions and objectives.	Page 10 to 14
**DISCUSSION**			
Summary of evidence	19	Summarize the main results (including an overview of concepts, themes, and types of evidence available), link to the review questions and objectives, and consider the relevance to key groups.	Page 14

SECTION	ITEM	PRISMA-ScR CHECKLIST ITEM	REPORTED ON PAGE #
Limitations	20	Discuss the limitations of the scoping review process.	Page 18
Conclusions	21	Provide a general interpretation of the results with respect to the review questions and objectives, as well as potential implications and/or next steps.	Page 19
**FUNDING**			
Funding	22	Describe sources of funding for the included sources of evidence, as well as sources of funding for the scoping review. Describe the role of the funders of the scoping review.	Page 20

JBI = Joanna Briggs Institute; PRISMA-ScR = Preferred Reporting Items for Systematic reviews and Meta-Analyses extension for Scoping Reviews.
* Where *sources of evidence* (see second footnote) are compiled from, such as bibliographic databases, social media platforms, and Web sites.
† A more inclusive/heterogeneous term used to account for the different types of evidence or data sources (e.g., quantitative and/or qualitative research, expert opinion, and policy documents) that may be eligible in a scoping review as opposed to only studies. This is not to be confused with *information sources* (see first footnote).
‡ The frameworks by Arksey and O'Malley (6) and Levac and colleagues (7) and the JBI guidance (4, 5) refer to the process of data extraction in a scoping review as data charting.
§ The process of systematically examining research evidence to assess its validity, results, and relevance before using it to inform a decision. This term is used for items 12 and 19 instead of "risk of bias" (which is more applicable to systematic reviews of interventions) to include and acknowledge the various sources of evidence that may be used in a scoping review (e.g., quantitative and/or qualitative research, expert opinion, and policy document).

From: Tricco AC, Lillie E, Zarin W, O'Brien KK, Colquhoun H, Levac D, et al. PRISMA Extension for Scoping Reviews (PRISMAScR): Checklist and Explanation. Ann Intern Med. 2018;169:467–473. doi: 10.7326/M18-0850.

Table S2. Search of all strategy

PUBMED

Search Group	Search Terms
Physical Activity	sport* OR sports[mesh] OR sports OR "motor activity"[mesh] OR "motor activity" OR "physical activity" OR "physical activit*" OR exercise[mesh] OR exercise OR "exercise*" OR "physical exercise*" OR "exercise program*" OR "physical education" OR "physical fitness"[mesh] OR "physical fitness" OR "leisure time" OR "leisure activit*" OR "aerobic activity" OR "physical inactivity")
Sedentary Behavior	sedentarism OR sedentary OR "sedentary behavior" OR "sedentary behaviors" OR "sedentary behaviour" OR "sedentary behaviours" OR "sedentary lifestyle*" OR "sedentary lifestyle"[mesh] OR "sedentary lifestyle" OR television[mesh] OR television OR "television time" OR "television watch*" OR "TV watch*" OR "screen time" OR "screen viewing" OR "screen media" OR "media screen time" OR "time sitting" OR sitting OR "sitting time" OR computers[mesh] OR computers OR "computer time" OR "computer use" OR "video game*"
Diet Behavior	diet[mesh] OR diet OR "diet behavior" OR "diet behaviour" OR "diet consumption" OR "dietary intake" OR "unhealthy diet" OR "healthy diet"[mesh] OR "healthy diet" OR nutrition OR "food behavior" OR "feeding behavior"[mesh] OR "feeding behavior" OR "feeding behaviors" OR "feeding behaviour" OR "feeding behaviours" OR "eating behavior" OR "eating behaviors" OR "eating behaviour" OR "eating behaviours" OR "food consumption" OR "food choice" OR "food intake" OR "food habit" OR "food habits" OR "food preferences"[mesh] OR "food preferences" OR "unhealthy food" OR "nutritional quality"
Analysis	"cluster analysis"[mesh] OR "cluster analysis" OR cluster OR cluster* OR clustering OR co-occur OR co-occurrence OR "behavior pattern" OR "behavior patterns" OR "behaviour pattern" OR "behaviour patterns" OR "lifestyle pattern" OR "lifestyle patterns" OR "latent class" OR "factor analysis" OR "factorial analysis" OR simultaneity
Population	youth OR adolesce* OR adolescent[mesh] OR adolescent OR adolescent* OR adolescence OR student* OR students[mesh] OR students OR teen* OR teenage* OR schoolchildren OR child* OR child[mesh] OR child OR children[mesh] OR children

Web of Science

**Search Group**	**Search Terms**
Physical Activity	TS=(sport* OR sports OR "motor activity" OR "physical activity" OR "physical activit*" OR exercise OR "exercise*" OR "physical exercise*" OR "exercise program*" OR "physical education" OR "physical fitness" OR "leisure time" OR "leisure activit*" OR "aerobic activity" OR recreation OR "physical inactivity")
Sedentary Behavior	TS=(sedentarism OR sedentary OR "sedentary behavior" OR "sedentary behaviors" OR "sedentary behaviour" OR "sedentary behaviours" OR "sedentary lifestyle*" OR "sedentary lifestyle" OR television OR "television time" OR "television watch*" OR "TV watch*" OR "screen time" OR "screen viewing" OR "screen media" OR "media screen time" OR "time sitting" OR sitting OR "sitting time" OR computers OR "computer time" OR "computer use" OR "video game*")
Diet Behavior	TS=(diet OR "diet behavior" OR "diet behaviour" OR "diet consumption" OR "dietary intake" OR "unhealthy diet" OR "healthy diet" OR nutrition OR "food behavior" OR "feeding behavior" OR "feeding behaviors" OR "feeding behaviour" OR "feeding behaviours" OR "eating behavior" OR "eating behaviors" OR "eating behaviour" OR "eating behaviours" OR "food consumption" OR "food choice" OR "food intake" OR "food habit" OR "food habits" OR "food preferences" OR "unhealthy food" OR "nutritional quality")
Analysis	TS=("cluster analysis" OR cluster OR cluster* OR clustering OR co-occur OR co-occurrence OR "behavior pattern" OR "behavior patterns" OR "behaviour pattern" OR "behaviour patterns" OR "lifestyle pattern" OR "lifestyle patterns" OR "latent class" OR "factor analysis" OR "factorial analysis" OR simultaneity)
Population	TS=(youth OR adolesce* OR adolescent OR adolescent* OR adolescence OR student* OR students OR teen* OR teenage* OR schoolchildren OR child* OR child OR children)

SCOPUS

**Search Group**	**Search Terms**
Physical Activity	TITLE-ABS-KEY(sport* OR sports OR "motor activity" OR "physical activity" OR "physical activit*" OR exercise OR "exercise*" OR "physical exercise*" OR "exercise program*" OR "physical education" OR "physical fitness" OR "leisure time" OR "leisure activit*" OR "aerobic activity" OR recreation OR "physical inactivity")
Sedentary Behavior	TITLE-ABS-KEY(sedentarism OR sedentary OR "sedentary behavior" OR "sedentary behaviors" OR "sedentary behaviour" OR "sedentary behaviours" OR "sedentary lifestyle*" OR "sedentary lifestyle" OR television OR "television time" OR "television watch*" OR "TV watch*" OR "screen time" OR "screen viewing" OR "screen media" OR "media screen time" OR "time sitting" OR sitting OR "sitting time" OR computers OR "computer time" OR "computer use" OR "video game*")
Diet Behavior	TITLE-ABS-KEY(diet OR "diet behavior" OR "diet behaviour" OR "diet consumption" OR "dietary intake" OR "unhealthy diet" OR "healthy diet" OR nutrition OR "food behavior" OR "feeding behavior" OR "feeding behaviors" OR "feeding behaviour" OR "feeding behaviours" OR "eating behavior" OR "eating behaviors" OR "eating behaviour" OR "eating behaviours" OR "food consumption" OR "food choice" OR "food intake" OR "food habit" OR "food habits" OR "food preferences" OR "unhealthy food" OR "nutritional quality")
Analysis	TITLE-ABS-KEY("cluster analysis" OR cluster OR cluster* OR clustering OR co-occur OR co-occurrence OR "behavior pattern" OR "behavior patterns" OR "behaviour pattern" OR "behaviour patterns" OR "lifestyle pattern" OR "lifestyle patterns" OR "latent class" OR "factor analysis" OR "factorial analysis" OR simultaneity)
Population	TITLE-ABS-KEY(youth OR adolesce* OR adolescent OR adolescent* OR adolescence OR student* OR students OR teen* OR teenage* OR schoolchildren OR child* OR child OR children)

LILACS, MEDLINE AND PSYCINFO

Search Group	Search Terms
Physical Activity	(sport OR sports OR "motor activity" OR "physical activity" OR "physical activities" OR exercise OR exercises OR "physical exercise" OR "exercise program*" OR "physical education" OR "physical fitness" OR "leisure time" OR "leisure activity" OR "leisure activities" OR "aerobic activity" OR recreation OR "physical inactivity")
Sedentary Behavior	(sedentarism OR sedentary OR "sedentary behavior" OR "sedentary behaviors" OR "sedentary behaviour" OR "sedentary behaviours" OR "sedentary lifestyles" OR "sedentary lifestyle" OR television OR "television time" OR "television watch" OR "television watches" OR "TV watch" OR "TV watching" OR "TC watches" OR "screen time" OR "screen viewing" OR "screen media" OR "media screen time" OR "time sitting" OR sitting OR "sitting time" OR computers OR "computer time" OR "computer use" OR "video game" OR "video games")
Diet Behavior	(diet OR "diet behavior" OR "diet behaviour" OR "diet consumption" OR "dietary intake" OR "unhealthy diet" OR "healthy diet" OR nutrition OR "food behavior" OR "feeding behavior" OR "feeding behaviors" OR "feeding behaviour" OR "feeding behaviours" OR "eating behavior" OR "eating behaviors" OR "eating behaviour" OR "eating behaviours" OR "food consumption" OR "food choice" OR "food intake" OR "food habit" OR "food habits" OR "food preferences" OR "unhealthy food" OR "nutritional quality")
Clustering	("cluster analysis" OR cluster OR cluster* OR clustering OR co-occur OR co-occurrence OR "behavior pattern" OR "behavior patterns" OR "behaviour pattern" OR "behaviour patterns" OR "lifestyle pattern" OR "lifestyle patterns" OR "latent class" OR "factor analysis" OR "factorial analysis" OR simultaneity)
Population	(youth OR adolesce* OR adolescent OR adolescent* OR adolescence OR student* OR students OR teen* OR teenage* OR schoolchildren OR child* OR child OR children)

Table S3. Behaviors included in clustering procedures along with physical activity, diet, and sedentary behavior.

**Author (year)**	**Behaviors included**
Androutsos et al. (2014)	Sleep.
Azeredo et al. (2016)	Aggression (intimidated and involved in a fight last 30d); Alcohol (frequency and glasses last 30d); Smoking (days last 30d); Drugs (use on last 30d); Unsafe sex (different people, use condom).
Bel-Serrat (2013)	None.
Berlin (2017)	Lose/gain weight.
Boone-Heinonen (2008)	Use of a community recreation center; Alcohol use; Smoking; Dieting or exercising to lose weight.
Busch (2013)	Alcohol use; Drug use; Smoking; Sexual behavior; Peer bullying.
Cameron (2011)	None.
Collese (2018)	Sleep time.
Cuenca-Garcia (2013)	None.
de Moraes (2016)	Sleep time.
Dantas (2018)	None.
Dumuid (2017)	None.
Dumuid (2017)	None.
Dumuid (2018)	Sleep.
Fernandez-Alvira (2013)	Sleep duration.
Ferrar (2015)	Sleep; Grooming; School activities.
Fleary (2017)	Unhealthy weight control; Sleep; Substance use (smoking, alcohol, binge drinking and marijuana)
Gubbels (2012)	None.
Gubbels (2012)	None.
Hartz (2018)	None.
Huh (2011)	Weight perception; Attitudes towards weight.
Iannotti (2013)	None.
Juresa (2012)	Family structure; Socio economic; Demographic characteristics; Alcohol and smoking; Teeth hygiene; Traffic safety; Physical conflicts; Health problems; Status and symptoms; Family medical history.
Kontogianni (2010)	None.
Lazzeri (2018)	Smoking; Alcohol; Violent behavior.
Laxer (2017)	Smoking; Alchool; Marijuana.
Laxer (2018)	Smoking; Alchool; Marijuana.
Leech (2014)	None.
Leech (2015)	None.
Lioret (2008)	None.
Magee et al. (2013)	Sleep duration; Sleeping/napping.

Maia et al. (2018)	Eating with the parents/guardians; Eating in front of the TV or studying; Having breakfast.
Mandic et al. (2017)	Body Weight.
Marttila-Tornio (2019)	Alcohol use; Smoking
Matias (2018)	None.
Matias (2018)	None.
Miguel-Berges (2017)	Sleep.
Moreira (2018)	None.
Moschonis (2013)	Sleep
Moschonis (2012)	Sleep
Nuutinen (2017)	Sleep quality
Ottevaere (2011)	None.
Pereira (2015)	Sleep
Perez-Rodrigo (2015)	Sleep
Platat (2006)	None.
Riggs (2012)	Weight consciousness; Weight loss attempts; Exercising to lose weight.
Rodrigues (2017)	Father and mother PA level; Father and mother excess weight.
Santaliestra-Pasias (2015)	None.
Seghers (2010)	None.
Sena (2017)	Consumption of alcoholic beverages; Tobacco experimentation.
Spengler (2012)	None.
Spengler (2014)	None.
Turner (2011)	Alcohol.
Van der Sluis (2010)	None.
Veloso (2012)	None.
Yen (2006)	Smoking; Drinking alcohol; Chewing betel nut were; Brushing teeth before bed; Staying up late; Hitting others; Swearing; Breaking things when angry; Vandalism; Stealing; Cheating on an examination; Washing hands before eating.

Table S4. Methodological Characteristics of included studies.

First Author	Study (year)	Sample Country	Sample (% of girls)	Age (range or mean)	PA (instrument)	Diet (instrument)	SB (instrument)	N. of behaviors	Cluster analysis
**High-Income Countries**									
Androutsos (2014)[1]	Healthy Growth Study (2007)	Greece	2.656 (50.1)	9-13	Questionnaire (Undefined)	24 hours recall	Questionnaire (Undefined)	12	PCA
Bel-Serrat (2013)[2]	IDEFICS (2007–2008)	Belgium, Cyprus, Estonia, Germany, Hungary, Italy, Spain, and Sweden.	4.619 (48.4)	2-9	Questionnaire (Undefined-Reproductible)	Questionnaire (Undefined-Reproductible)	Questionnaire (Undefined-Reproductible)	4	K-means*
Berlin (2017)[3]	ECLS-K (1998-1999)	U.S.A.	9.295 (49)	13-15	Questionnaire (Undefined)	Questionnaire (Undefined)	Questionnaire (Undefined)	21	LPA
Boone-Heinonen (2008)[4]	National Longitudinal Study of Adolescent Health (1995 and 1996)	U.S.A.	8.840 (50.7)	11-21	Questionnaire (Sallis et al., 1999)	Questionnaire (Sallis et al., 1999)	Questionnaire (Sallis et al., 1999)	37	K-means
Busch (2013)[5]	Not informed (2012)	Netherlands	2.690 (55.0)	11-18	Questionnaire (adapted from the Dutch version of HBSC)	Questionnaire (adapted from the Dutch version of HBSC)	Questionnaire (adapted from the Dutch version of HBSC)	15	PCA & Two-steps

Cameron (2012)[6]	READI (2007-2008)	Australia	352	5-12	Objective Measure (Accelerometer)	Questionnaire (Undefined)	Questionnaire (Undefined)	4	Ward
Cuenca-García (2013)[7]	HELENA (2006-2007)	Austria, Belgium, France, Greece, Germany, Hungary, Italy, Spain, and Sweden.	2.084 (54.0)	12-14	Questionnaire (IPAQ-A)	24 hours recall	Questionnaire (Undefined-Reproductible)	4	K-means*
Dumuid (2017)[8]	ISCOLE (2011-2012)	Australia	284 (53.9)	9-11	Objective Measure (Accelerometer)	Questionnaire (FFQ)	Questionnaire (Undefined)	7	K-means*
Fernández-Alvira (2013)[9]	ENERGY-project (2010)	Belgium, Greece, Hungary, Netherlands, Norway, Slovenia, and Spain.	5.284 (54.0)	10-12	Questionnaire (ENERGY-child)	Questionnaire (ENERGY-child)	Questionnaire (ENERGY-child)	4	K-means*
Ferrar and Golley (2015)[10]	National Children’s Nutrition and Physical Activity Survey (2007)	Australia	1.853 (49.8)	9-16	Questionnaire (Multimedia Activity Recall for Children and Adults)	Questionnaire (Food Model Booklet)	Questionnaire (Multimedia Activity Recall for Children and Adults)	24	Two-steps

Fleary (2017)[11]	YRBSS (2011)	U.S.A.	14.815 (49.0)	NR	Questionnaire (Undefined-Reproductible)	Questionnaire (Undefined-Reproductible)	Questionnaire (Undefined-Reproductible)	15	LCA
Gubbels (2011)[12]	The KOALA Birth Cohort Study (2000)	Netherlands	2.074 (48.6)	5	Questionnaire (Standard Questionnaire for measuring Physical Activity)	Questionnaire (FFQ)	Questionnaire (Standard Questionnaire for measuring Physical Activity)	30	PCA
Gubbels (2012)[13]	The KOALA Birth Cohort Study (2000)	Netherlands	2.074 (48.6)	5	Questionnaire (Standard Questionnaire for measuring Physical Activity)	Questionnaire (FFQ)	Questionnaire (Standard Questionnaire for measuring Physical Activity)	13	PCA
Hartz (2018)[14]	NHANES (2003-2004)	U.S.A.	1.233 (48.5)	12-19	Objective Measure (Accelerometer)	24 hours recall	Objective Measure (Accelerometer)	3	LCA
Huh et al (2011)[15]	Pathways Study (Not informed)	U.S.A.	997 (48.1)	Mean: 9.6	Questionnaire (Undefined)	Questionnaire (Undefined)	Questionnaire (Undefined)	11	LCA
Iannotti and Wang (2013)[16]	Not informed (Not informed)	U.S.A.	9.206 (51.6)	Mean: 13.9	Questionnaire (Undefined-Reproductible)	Questionnaire (FFQ)	Questionnaire (Undefined-Reproductible)	10	LCA
Kontogianni (2010)[17]	Not informed (2007)	Greece	Children: 751 (49.0) Adolescents: 554 (56.0)	Children: 3-12 Adolescents: 13-18	Questionnaire (Undefined)	24 hours recall	Questionnaire (Undefined)	9	PCA
Landsberg (2010)[18]	KOPS (1996-2006)	Germany	1.894 (51.5)	Mean: 14.7	Questionnaire (Undefined)	Questionnaire (FFQ)	Questionnaire (Undefined)	7	Two-steps
Laxer (2017)[19]	COMPASS (2012-2013)	Canada	18.587 (48.9)	9-12	Questionnaire (Undefined-Reproductible)	Questionnaire (Undefined-Reproductible)	Questionnaire (Undefined-Reproductible)	15	LCA
Laxer (2018)[20]	COMPASS (2012-2013)	Canada	5.084 (52.1)	Mean: 14.7	Questionnaire (Undefined-Reproductible)	Questionnaire (Undefined-Reproductible)	Questionnaire (Undefined-Reproductible)	15	LCA

Lazzeri (2018)[21]	HBSC (2010)	Italy	3.291	11, 13 and 15	Questionnaire (HBSC)	Questionnaire (HBSC)	Questionnaire (HBSC)	22	EFA & K-means
Leech (2014)[22]	HEAPS (2002-2003)	Australia	Younger children: 362 (50.0) Older children: 610 (56.0)	Younger children: 5-6 Older children: 10-12	Objective Measure (Accelerometer)	Questionnaire (FFQ)	Objective Measure (Accelerometer)	5	K-means
Leech (2015)[23]	HEAPS (2002-2003)	Australia	Younger children: 123 (46.0) Older children: 87 (43.0)	Younger children: 5-6 Older children: 10, 12 & 14	Objective Measure (Accelerometer)	Questionnaire (FFQ)	Objective Measure (Accelerometer)	5	K-means
Lioret (2008)[24]	French INCA1 (1998-1999)	France	748	3-11	Questionnaire (Modifiable Activity Questionnaire)	7 days recall	Questionnaire (Modifiable Activity Questionnaire)	34	PCA
Magee (2013)[25]	LSAC (2006-2008)	Australia	1.833 (48.4)	6-9	Diares	Diares	Diares	6	LCA
Mandic (2017)[26]	BEATS (2014-2015)	New Zeland	1.300 (51.0)	13-18	Questionnaire (HBSC)	Questionnaire (HBSC)	Questionnaire (HBSC)	4	Two-steps
Marttila-Tornio (2019)[27]	Northen Finland Birth Cohort 1986	Finland	4.305 (53.0)	15-16	Questionnaire (NFBC1986)	Questionnaire (NFBC1986)	Questionnaire (NFBC1986)	7	K-means
Miguel-Berges (2017)[28]	ToyBox (2012)	Belgium, Bulgaria, Germany, Greece,	5.387 (49.0)	3.5-5.5	Objective Measure (Pedometer) & Questionnaire (Primary Caregivers Questionnaire)	Questionnaire (Primary Caregivers Questionnaire)	Questionnaire (Primary Caregivers Questionnaire)	6	K-means*

		Poland, and Spain							
Moschonis (2013)[29]	Healthy Growth Study (2007)	Greece	2.043 (50.2)	9-13	Questionnaire (Manios, Kafatos & Markakis, 1998)	24 hours recall	Questionnaire (Undefined)	12	PCA
Moschonis (2014)[30]	Healthy Growth Study (2007)	Greece	2.073 (50.2)	9-13	Questionnaire (Manios, Kafatos & Markakis, 1998)	24 hours recall	Questionnaire (Undefined)	12	PCA
Nuutinen (2017)[31]	HBSC (2010)	Finland	13 years: 2.152<br>15 years: 2.110	13 and 15	Questionnaire (HBSC)	Questionnaire (HBSC)	Questionnaire (HBSC)	9	K-means
Ottevaere (2011)[32]	HELENA (2006-2007)	Belgium, France, Greece, Germany, Italy, Spain, and Sweden.	2.084 (54.4)	Youngers: 12.5-14.9<br>Olders: 15-17.5	Questionnaire (IPAQ-A)	24 hours recall	Questionnaire (HELENA questionnaire)	3	K-means
Pereira (2015)[33]	ISCOLE (2011-2012)	Portugal	686 (55.5)	9.5-10.5	Objective Measure (Accelerometer)	Questionnaire (FFQ)	Questionnaire (YRBSS)	5	LCA
Pérez-Rodrigo (2015)[34]	ANIBES Study (2012)	Spain	415 (37.8)	9-17	Questionnaire (IPAQ-A)	24 hours recall	Questionnaire (HELENA questionnaire)	4	K-means*
Platat (2006)[35]	Not informed (2001)	France	2.724	12	Questionnaire (Modifiable Activity Questionnaire)	Questionnaire (Undefined)	Questionnaire (Modifiable Activity Questionnaire)	6	MCA
Riggs (2012)[36]	Pathways (Not informed)	U.S.A.	997 (51.9)	Mean: 9.3	Questionnaire (Undefined)	Questionnaire (Undefined)	Questionnaire (Undefined)	12	LCA

Rodrigues (2017)[37]	PPSOC (2009-2010)	Portugal	10.258 (51.0)	6-9	Questionnaire (Undefined-Reproductible)	Questionnaire (Undefined-Reproductible)	Questionnaire (Undefined-Reproductible)	15	PCA
Santaliestra-Pasías (2015)[38]	IDEFICS (2007-2008)	Belgium, Cyprus, Estonia, Germany, Hungary, Italy, Spain, and Sweden.	11.674 (49.2)	2-9	Questionnaire (Undefined-Reproductible)	Questionnaire (CEHQ-FFQ)	Questionnaire (Undefined-Reproductible)	4	K-means*
Seghers and Rutten (2010)[39]	Not informed (2007)	Belgium	317 (56.8)	Mean: 11.7	Questionnaire (Flemish Physical Activity Questionnaire)	Questionnaire (FFQ)	Questionnaire (Undefined)	5	K-means
Spengler (2012)[40]	KIGGS MoMo (2003-2006)	Germany	1.643 (49.4)	11-17	Questionnaire (MoMo-PAQ overall activity index)	Questionnaire (Adapted FFQ)	Questionnaire (Undefined)	3	K-means*
Spengler (2014)[41]	KIGGS MoMo (2003-2006)	Germany	1.642 (49.4)	11-17	Questionnaire (MoMo-PAQ overall activity index)	Questionnaire (Adapted FFQ)	Questionnaire (Undefined)	3	K-means*
Turner (2011)[42]	Not informed (2005)	Canada	445 (60.2)	14-17	Questionnaire (SAPAC)	Questionnaire (adapted from the WHO Steps instrument)	Questionnaire (SAPAC)	7	Two-step
Van der Sluis (2010)[43]	Fruits and Vegetables Make the Marks (2001-2005)	Norway	713 (53.0)	Not reported	Questionnaire (Undefined-Reproductible)	Questionnaire (Andersen et al., 2004)	Questionnaire (Undefined-Reproductible)	4	K-means

Veloso (2012)[44]	HBSC (2010)	Portugal	3.069 (54.1)	13-16.9	Questionnaire (HBSC)	Questionnaire (HBSC)	Questionnaire (HBSC)	8	K-means
Yen (2006)[45]	CABLE (2001)	Taiwan	2.075 (48.0)	Not reported	Questionnaire (CABLE)	Questionnaire (CABLE)	Questionnaire (CABLE)	18	PCA
**Upper-Middle Income Countries**									
Azeredo (2016)[46]	National Survey of School Health (2015)	Brazil	104.109 (52.2)	13-16	Questionnaire (PeNSE Questionnaire)	Questionnaire (PeNSE Questionnaire)	Questionnaire (PeNSE Questionnaire)	18	FA
Collese (2018)[47]	HELENA (2006-2007) BRACAH (2007)	Brazil	HELENA study: 1,252 (52.7) BRACAH study: 682 (54.2)	HELENA: 12.5–17.5 years (14.7) BRACAH: 14 to 17.5 years (16.3)	Questionnaire (IPAQ-A)	Questionnaire (FFQ)	Questionnaire (HELENA questionnaire)	5	K-means*
Dantas (2018)[48]	Health Education Program through Dietary Interventions and Physical Activity (2017)	Brazil	578 (67.8)	12–18	Questionnaire (IPAQ-A)	Questionnaire (YRBSS)	Questionnaire (Undefined-Reproductible)	4	K-means*
Juresa (2012)[49]	School Health Survey	Croatia	960 (48.6)	Mean: 7.5	Questionnaire (School Health Survey)	Questionnaire (School Health Survey)	Questionnaire (School Health Survey)	41	EFA

	(2003-2004)								
Maia (2018)[50]	PeNSE (2012)	Brazil	109.104 (52.2)	Younger than 13 and older tha 16	Questionnaire (PeNSE Questionnaire)	Questionnaire (PeNSE Questionnaire)	Questionnaire (PeNSE Questionnaire)	17	K-means
Matias (2018)[51]	PeNSE (2012)	Brazil	102.072	11-19	Questionnaire (PeNSE Questionnaire)	Questionnaire (PeNSE Questionnaire)	Questionnaire (PeNSE Questionnaire)	4	Two-steps
Matias (2018)[52]	PeNSE (2012)	Brazil	102.072 (51.3)	11-9 (14.3)	Questionnaire (PeNSE Questionnaire)	Questionnaire (PeNSE Questionnaire)	Questionnaire (PeNSE Questionnaire)	4	Two-steps
Sena (2017)[53]	Not informed (2009-2011)	Brazil	1.716 (49.3)	10-17	Questionnaire (Undefined)	Questionnaire (FFQ)	Questionnaire (Undefined)	6	PCA
**Intercontinental Studies with Different Income Countries**									
Dumuid (2016)[54]	ISCOLE (2011-2012)	Australia, Brazil, Canada, China, Colombia, England, Finland, India, Kenya, Portugal, South Africa, and USA	5.710	9-11	Objective Measure (Accelerometer)	Questionnaire (FFQ)	Objective Measure (Accelerometer)	9	K-means*
Dumuid (2017)[55]	ISCOLE (2011-2012)	Australia, Brazil,	5.759 (55.0)	9-11	Objective Measure (Accelerometer)	Questionnaire (FFQ)	Questionnaire (Undefined)	8	K-means*

		Canada, China, Colombia, England, Finland, India, Kenya, Portugal, South Africa, and USA.							
Moraes (2016)[56]	HELENA (2006-2007) BRACAH (2007)	HELENA: Austria, Belgium, France, Greece, Germany, Hungary, Italy, Spain, and Sweden. BRACAH: Brazil.	HELENA study: 1.252 (52.7) BRACAH study: 682 (54.3)	HELENA: 12-17.5 BRACAH: 14-17.5	Questionnaire (IPAQ-A)	Questionnaire (HBSC)	Questionnaire (Undefined)	5	K-means*
Moreira (2018)[57]	HELENA (2006-2007) BRACAH (2007)	HELENA: Austria, Belgium, France,	HELENA study: 2.057 (53.8) ELANA study: 968 (53.2)	HELENA: 12.5-17.7 ELANA: 13.5-19	HELENA: Questionnaire (IPAQ-A) ELANA: Questionnaire (IPAQ short version)	HELENA: 24 hours recall ELANA: Questionnaire (FFQ)	HELENA: Questionnaire (HELENA questionnaire) ELANA:	4	K-means*

Greece, Germany, Hungary, Italy, Spain, and Sweden. BRACAH: Brazil.	Questionnaire (Undefined)

PA: physical activity; SB: sedentary behavior; NA: not applicable; NR: not reported; *: With Wald's method; IDEFICS: Identification and prevention of Dietary and lifestyle-induced health Effects in children and InfantS; ECLS-K: Early Childhood Longitudinal Study Cohort; READI: Resilience for Eating and Activity Despite Inequality; HEAPS: Health, Eating and Play study; LSAC: Longitudinal Study of Australian Children; BEATS: Built Environment and Active Transport to School; Pathways: Pathways to Health; PPSOC: Portuguese Prevalence Study of Obesity in Childhood; BRACAH: Brazilian Cardiovascular Adolescent Health; CABLE: Child and Adolescent Behaviors in Long-term Evolution; ELANA: Adolescent Nutritional Assessment Longitudinal; ENERGY: EuropeaN Energy balance Research to prevent excessive weight Gain among Youth; FFQ: Food Frequency Questionnaire; HBSC: Health Behavior in School-aged Children; HELENA: Healthy Lifestyle in Europe by Nutrition in Adolescence; IPAQ-A: International Physical Activity Questionnaire for Adolescents; MoMo: Motorik-Modul; PeNSE: National School-based Health Survey; SAPAC: Self-Administered Physical Activity Checklist YRBSS: Youth Risk Behavior Surveillance System.

EFA: Exploratory factor analysis; FA: Factor analysis; LCA: Latent class analysis; LPA: Latent profile analysis; MCA: Multiple correspondence analysis; PCA: Principal component analysis.

Equal superscript black letters indicate common samples.

Table S5. Assessment of the bias risk of studies.

Study	Selection bias		Study design				Assessment tool: PA			Assessment tool: Diet			Assessment tool: SB			Withdrawals and drop-outs		
	Q1	Bias	Q2	Q3	Q4	Bias	Q5	Q6	Bias	Q5	Q6	Bias	Q5	Q6	Bias	Q7	Q8	Bias
Androutsos (2014)[1 a]	0	Moderate	1	1	1	Strong	0	0	Weak	1	1	Strong	0	1	Weak	1	1	Strong
Azeredo (2016)[46 b]	1	Strong	1	1	1	Strong	0	1	Weak	0	1	Weak	0	1	Weak	1	1	Strong
Bel-Serrat (2013)[2 a]	-1	Weak	1	1	0	Moderate	1	1	Strong	1	1	Strong	0	1	Weak	1	1	Strong
Berlin (2017)[3 a]	?	Weak	1	0	0	Moderate	0	1	Weak	0	1	Weak	0	1	Weak	0	1	Strong
Boone-Heinonen (2008)[4 a]	0	Moderate	1	1	0	Moderate	1	1	Strong	1	1	Strong	1	1	Strong	1	0	Moderate
Busch (2013)[5 a]	0	Moderate	0	1	-1	Weak	?	1	Weak	?	1	Weak	?	1	Weak	1	1	Strong
Cameron (2012)[6 a]	-1	Weak	0	1	1	Moderate	1	1	Strong	0	1	Weak	0	1	Weak	1	-1	Weak
Collese (2018)[47 b]	-1	Weak	1	1	1	Strong	1	1	Strong	1	1	Strong	1	1	Strong	0	0/?	Weak
Cuenca-García (2013)[7 a]	-1	Weak	0	0	0	Weak	1	1	Strong	1	1	Strong	1	1	Strong	0	-1	Weak
Dantas (2018)[48 b]	-1	Weak	0	1	1	Moderate	1	1	Strong	1	1	Strong	0	1	Weak	0	0	Weak
Dumuid (2017)[55]	0	Moderate	0	1	*	Weak	1	1	Strong	1	1	Strong	0	1	Weak	1	1	Strong
Dumuid (2017)[8 c]	-1	Weak	0	1	1	Moderate	1	1	Strong	1	1	Strong	0	1	Weak	1	-1	Weak
Dumuid (2016)[54 c]	1	Strong	1	1	1	Strong	1	1	Strong	1	1	Strong	1	1	Strong	1	0	Moderate
Fernández-Alvira (2013)[9 a]	0	Moderate	1	1	1	Strong	0	1	Weak	0	1	Weak	0	1	Weak	0	1	Strong
Ferrar and Golley (2015)[10 a]	1	Strong	0	-1	1	Weak	1	1	Strong	0	1	Weak	1	1	Strong	0	1	Strong
Fleary (2017)[11 a]	?	Weak	1	0	0	Moderate	0	1	Weak	0	1	Weak	0	1	Weak	1	1	Strong
Gubbels (2011)[12 a]	0	Moderate	0	1	-1	Weak	0	1	Weak	1	1	Strong	0	1	Weak	0	0	Moderate
Gubbels (2012)[13 a]	0	Moderate	0	1	-1	Weak	0	1	Weak	1	1	Strong	0	1	Weak	0	0	Moderate
Hartz (2018)[14 a]	-1	Weak	1	1	1	Strong	1	1	Strong	0	1	Weak	1	1	Strong	1	0	Moderate

Huh et al (2011)[15] [a]	?	Weak	0	-1	1	Weak	0	0	Weak	0	1	Weak	0	0	Weak	1	0	Moderate
Iannotti and Wang (2013)[16] [a]	1	Strong	1	1	1	Strong	1	1	Strong	1	1	Strong	1	1	Strong	1	1	Strong
Juresa (2012)[49] [b]	1	Strong	1	1	1	Strong	0	1	Weak	0	1	Weak	0	1	Weak	0	-1	Weak
Kontogianni (2010)[17] [a]	1	Strong	1	1	1	Strong	0	1	Weak	1	1	Strong	0	1	Weak	0	1	Strong
Landsberg (2010)[18] [a]	-1	Weak	1	1	1	Strong	1	1	Strong	1	1	Strong	1	1	Strong	1	-1	Weak
Laxer (2017)[19] [a]	-1	Weak	0	1	-1	Weak	1	1	Strong	1	1	Strong	1	1	Strong	1	1	Strong
Laxer (2018)[20] [a]	-1	Weak	0	1	-1	Weak	1	1	Strong	1	1	Strong	1	1	Strong	1	-1	Weak
Lazzeri (2018)[21] [a]	1	Strong	1	1	1	Strong	0	1	Weak	1	1	Strong	0	0	Weak	0	?	Weak
Leech (2014)[22] [a]	-1	Weak	1	1	1	Strong	1	1	Strong	1	1	Strong	1	1	Strong	1	0	Moderate
Leech (2015)[23] [a]	-1	Weak	1	1	1	Strong	1	1	Strong	1	1	Strong	1	1	Strong	1	-1	Weak
Lioret (2008)[24] [a]	-1	Weak	1	1	1	Strong	1	1	Strong	0	1	Weak	1	1	Strong	1	1	Strong
Magee (2013)[25] [a]	?	Weak	0	-1	1	Weak	0	1	Weak	0	1	Weak	0	1	Weak	0	?	Weak
Maia (2018)[50] [b]	1	Strong	1	1	1	Strong	0	1	Weak	0	1	Weak	0	1	Weak	1	1	Strong
Mandic (2017)[26] [a]	-1	Weak	0	1	0	Weak	1	1	Strong	1	1	Strong	1	1	Strong	1	0	Moderate
Marttila-Tornio (2019)[27] [a]	-1	Weak	1	1	-1	Weak	1	1	Strong	1	1	Strong	1	1	Strong	1	-1	Weak
Matias (2018)[51] [b]	1	Strong	1	1	1	Strong	0	1	Weak	0	1	Weak	0	1	Weak	1	1	Strong
Matias (2018)[52] body image[b]	1	Strong	1	1	1	Strong	1	1	Strong	0	1	Weak	0	1	Weak	1	1	Strong
Miguel-Berges (2017)[28] [a]	0	Moderate	0	1	1	Moderate	1	1	Strong	1	1	Strong	1	1	Strong	1	0	Moderate
Moraes (2016)[56] [c]	-1	Weak	1	1	1	Strong	1	1	Strong	1	1	Strong	1	1	Strong	1	0	Moderate
Moreira (2018)[57] [c]	1	Strong	1	1	1	Strong	1	1	Strong	1	1	Strong	1	1	Strong	1	-1	Weak
Moschonis (2013)[29] [a]	0	Moderate	1	1	1	Strong	1	1	Strong	1	1	Strong	1	1	Strong	1	0	Moderate
Moschonis (2014)[30] [a]	0	Moderate	1	1	1	Strong	1	1	Strong	1	1	Strong	1	1	Strong	1	0	Moderate
Nuutinen (2017)[31] [a]	1	Strong	1	1	1	Strong	1	1	Strong	1	1	Strong	1	1	Strong	0	1	Strong
Ottevaere (2011)[32] [a]	-1	Weak	1	1	1	Strong	1	1	Strong	1	1	Strong	1	1	Strong	1	-1	Weak

Pereira (2015)[33 a]	1	Strong	1	1	1	Strong	1	1	Strong	1	1	Strong	1	1	Strong	0	?	Weak
Pérez-Rodrigo (2015)[34 a]	1	Strong	1	1	1	Strong	1	1	Strong	1	1	Strong	1	1	Strong	1	-1	Weak
Platat (2006)[35 a]	0	Moderate	1	1	1	Strong	1	1	Strong	0	1	Weak	1	1	Strong	1	0	Moderate
Riggs (2012)[36 a]	?	Weak	1	1	1	Strong	1	1	Strong	0	1	Weak	0	1	Weak	0	1	Strong
Rodrigues (2017)[37 a]	-1	Weak	1	1	1	Strong	0	1	Weak	0	1	Weak	0	1	Weak	1	1	Strong
Santaliestra-Pasías (2015)[38 a]	0	Moderate	1	1	1	Strong	1	1	Strong	1	1	Strong	0	1	Weak	1	0	Moderate
Seghers and Rutten (2010)[39 a]	?	Weak	1	1	1	Strong	1	1	Strong	1	1	Strong	1	1	Strong	1	1	Strong
Sena (2017)[53 b]	0	Moderate	1	1	1	Strong	1	1	Strong	1	1	Strong	0	1	Weak	1	0	Moderate
Spengler (2012)[40 a]	?	Weak	1	1	1	Strong	1	1	Strong	1	1	Strong	1	1	Strong	1	1	Strong
Spengler (2014)[41 a]	?	Weak	1	1	1	Strong	1	1	Strong	1	1	Strong	1	1	Strong	1	0	Moderate
Turner (2011)[42 a]	?	Weak	0	1	-1	Weak	1	1	Strong	0	1	Weak	0	1	Weak	1	1	Strong
Van der Sluis (2010)[43 a]	1	Strong	1	1	1	Strong	0	1	Weak	1	1	Strong	0	1	Weak	1	1	Strong
Veloso (2012)[44 a]	0	Moderate	1	1	1	Strong	1	1	Strong	1	1	Strong	1	1	Strong	0	0	Moderate
Yen (2006)[45 a]	-1	Weak	1	1	1	Strong	1	1	Strong	1	1	Strong	1	1	Strong	1	1	Strong

PA: physical activity; SB: sedentary behavior; Q1: Are the individuals selected to participate in the study likely to be representative of the target population?; Q2: Is there a description of the representativeness of the sample?; Q3: Was the sampling method described?; Q4: Was the method appropriate?; Q5: Is there a prior validation report of the tool?; Q6: Is there information that makes it possible to replicate the tool?; Q7: Were withdrawals and drop-outs reported in terms of numbers and/or reasons per group?; Q8: Indicate the percentage of participants completing the study; ?: impossible to determine; *The selection method differed among study countries. [a]High-Income Countries; [b]Upper-Middle Income Countries; [c]Involves samples from more than one country and with different income classification (Intercontinental Studies with Different Income Countries).

Table S6. Articles from their respective studies.

1. ISCOLE (1)
    - Dumuid (2016)
    - Dumuid (2017)
    - **Dumuid (2017) – Intercontinental – 5.759 – 9-11 – Children & Adolescents (C&A) – Australia, Brazil, Canada, China, Colombia, Finland, India, Kenya, Portugal, South Africa, England, USA.**
    - Pereira (2015)
2. HBSC – 11, 13, 15 – Adolescents (A)
    - **Lazzeri (2018) - Italy**
    - **Nuutinen (2017) - Finland**
    - **Veloso (2012) - Portugal**
3. HELENA
    - **Cuenca-García (2013) – European Countries – 2.084 – 12-14 – A**
    - Moraes (2016)
    - Moreira (2018)
    - Ottevaere (2011)
    - Collese (2018)
4. Healthy Growth Study (2007)
    - **Androutsos (2014) – Greece – 2.656 – 9-13 – C&A**
    - Moschonis (2012)
    - Moschonis (2013)
5. HEAPS
    - **Leech (2014) – Australia – 972 – 5-6 & 10-12 – C&A**
    - Leech (2015)
6. IDEFICS
    - Bel-Serrat (2013)
    - **Santaliestra-Pasías (2015) – European Countries – 11.674 – 2-9 – Children (C)**
7. KIGGS/MoMo
    - **Spengler (2012) – Germany – 1.643 – 11-17 - A**
    - Spengler (2014)
8. KOALA Birth Cohort
    - **Gubbels (2011) – Netherlands – 2.074 – 5 – C**
    - Gubbels (2012)
9. Pathways to Health
    - **Huh (2011) – USA – 997 – 9.6 - C**
    - Riggs (2012)
10. PeNSE 2015
    - **Azeredo (2016) – Brazil – 104.109 – 13-16 or more - A**

- Matias (2018)
- Matias (2018) – Body Image

11. 2011 YRBSS
    - Fleary (2017) – USA – 14.815 – Not reported (NR)
12. ANIBES
    - Pérez-Rodrigo (2016) – Spain – 415 – 9-17 – C&A
13. BEATS
    - Mandic (2017) – New Zeland – 1.300 – 13-18 - A
14. BRACAH
    - **Moraes (2016) – Brazil – 682 – 14-17.5 – A**
    - Collese (2018)
15. CABLE
    - Yen (2006) – Taiwan – 2.075 - NR
16. COMPASS
    - **Laxer (2017) – Canada – 18.587 – 9-12 – C&A**
    - Laxer (2018)
17. ECLS-K
    - Berlin (2015) – USA – 9.295 – 13-15 - A
18. ELANA
    - Moreira (2018) – Brazil – 968 – 13.5-19 - A
19. ENERGY
    - Fernández-Alvira (2013) – European Countries – 5.284 – 10-12 - A
20. French INCA1
    - Lioret (2008) – France – 748 – 3-11 – C&A
21. Fruits & Vegetables Make the Marks
    - Van Der Sluis (2010) – Norway – 713 - NR
22. KOPS
    - Landsberg (2010) – Germany – 1.894 – 14.7 - A
23. LSAC
    - Magee (2013) – Australia – 1.833 – 6-9 - C
24. National Children’s Nutrition and PA Survey
    - Ferrar (2015) – Australia – 1.853 – 9-16 – C&A
25. National Longitudinal Study of Adolescent Health
    - Boone-Heinonen – USA – 8.840 – 11-21 - A
26. NHANES
    - Hartz (2018) – USA – 1.233 – 12-19 - A
27. PeNSE 2012
    - Maia (2017) – Brazil – 109.104 - <13 & >16 - A
28. PPSOC
    - Rodrigues (2017) – Portugal – 10.258 – 6-9 - C

29. READI
    - Cameron (2011) – Australia – 352 – 5-12 – C&A
30. School Health Survey
    - Juresa (2012) – Croatia – 960 – 7.5 - C
31. ToyBox
    - Miguel-Berges (2017) – European Countries – 5.387 – 3.5-5.5 - C
32. Article that did not presented the study name: 1
    - Busch (2013) – Netherlands – 2.690 – 11-18 - A
33. Article that did not presented the study name: 2
    - Iannotti (2013) – USA – 9.206 – 13.9 - A
34. Article that did not presented the study name: 3
    - Kontogianni (2010) – Greece – 1.305 – 3-18 – C&A
35. Article that did not presented the study name: 4
    - Platat (2006) – France – 2.724 – 12 - A
36. Article that did not presented the study name: 5
    - Seghers (2010) – Belgium – 317 – 11.7 - A
37. Article that did not presented the study name: 6
    - Sena (2017) – Brazil – 1.716 – 10-17 - A
38. Article that did not presented the study name: 7
    - Turner (2011) – Canada – 445 – 14-17 – A
39. Health Education Program through Dietary Interventions and Physical Activity
    - Dantas (2018) – Brazil – 578 – 12-18 – A
40. Northen Finland Birth Cohort 1986 (NFBC1986)
    - Marttila-Tornio (2019) – Finland – 4.305 – 15-16 – A

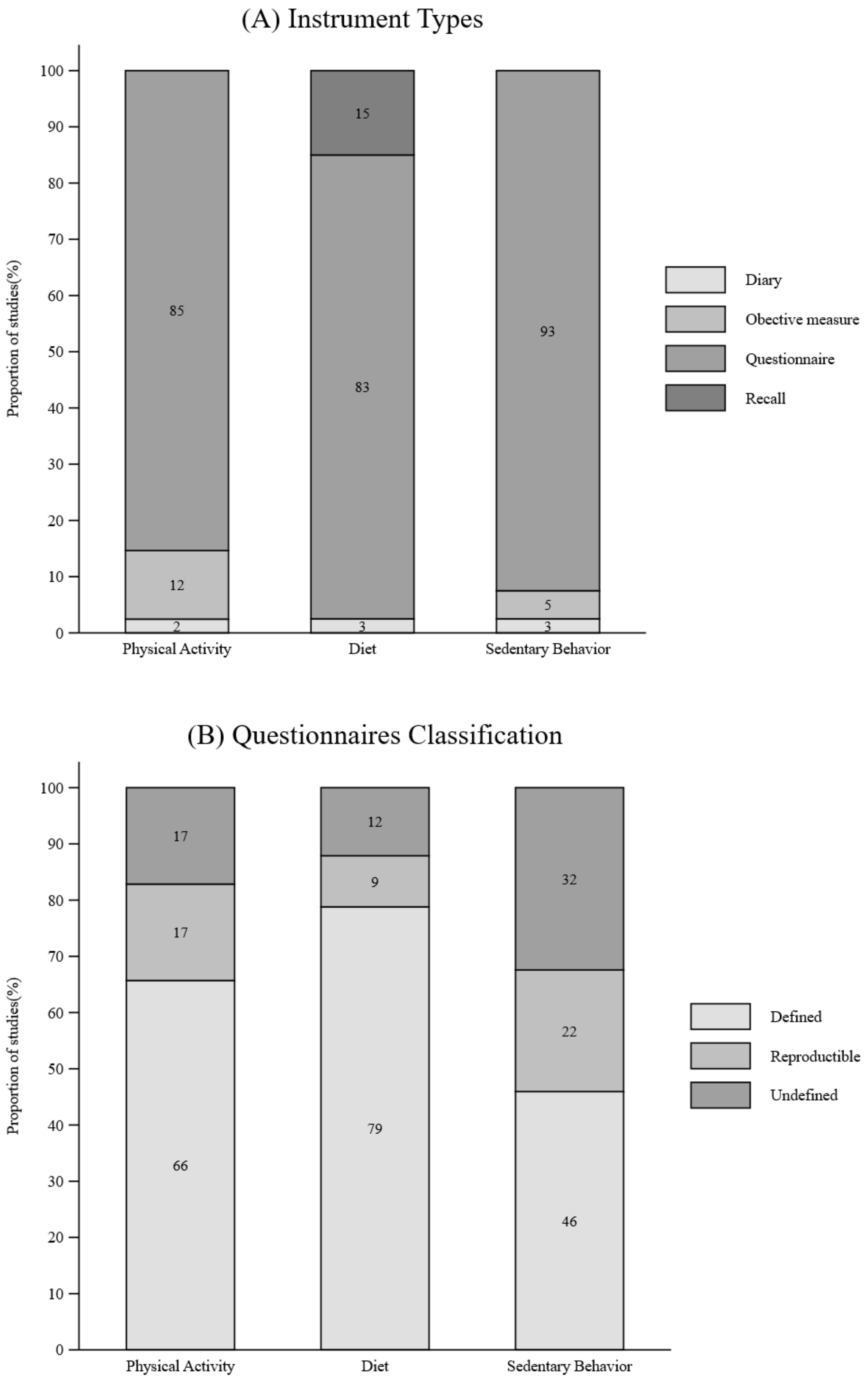

Figure S1. Instruments used and questionnaires classification according to each behavior.

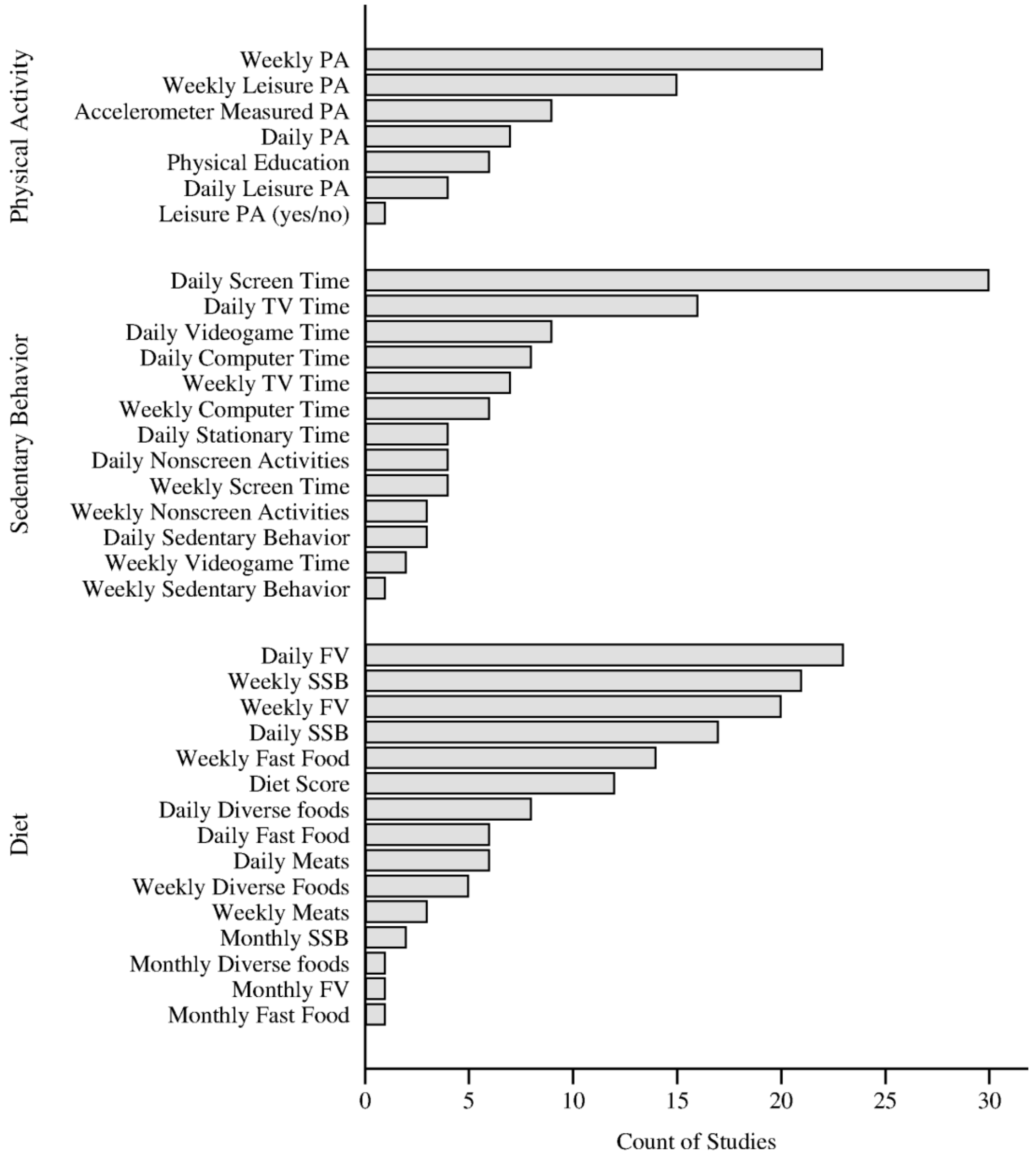

Figure S2. Behavioral outcomes used to define PA, diet, and sedentary behavior in clustering procedures.

Note: FV: fruits and/or vegetables; SSB: salty and sugary snacks, and/or sweetened beverages. *Stationary time refers to accelerometer measured movement behaviors.

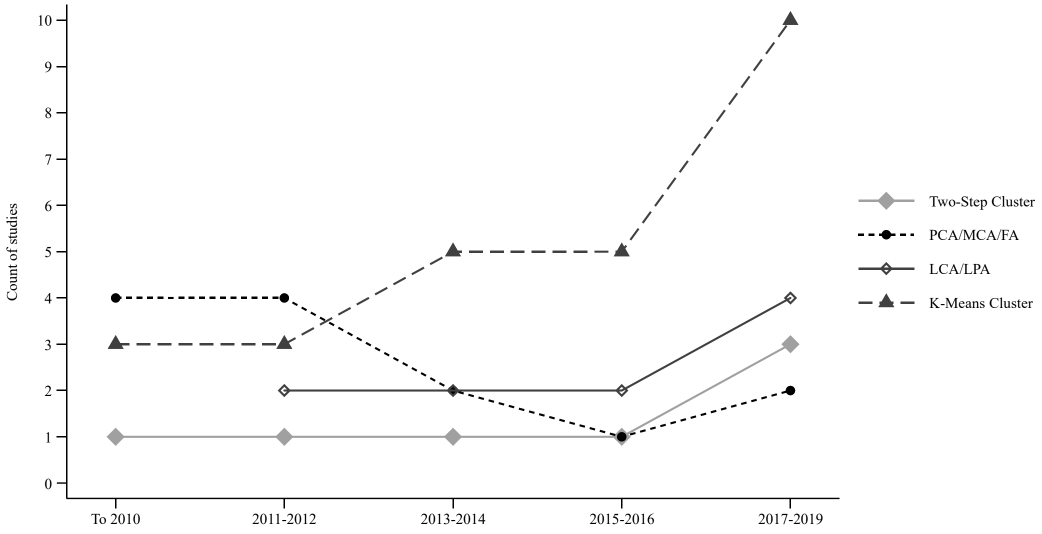

Figure S3. Use of statistical procedures to evaluate the clustering between physical activity, diet and sedentary behavior among children and adolescents.

Note: LCA: Latent Class Analysis; LPA: Latent Profile Analysis; PCA: Principal Component Analysis; MCA: Multiple corresponding analysis; FA: Factorial Analysis.

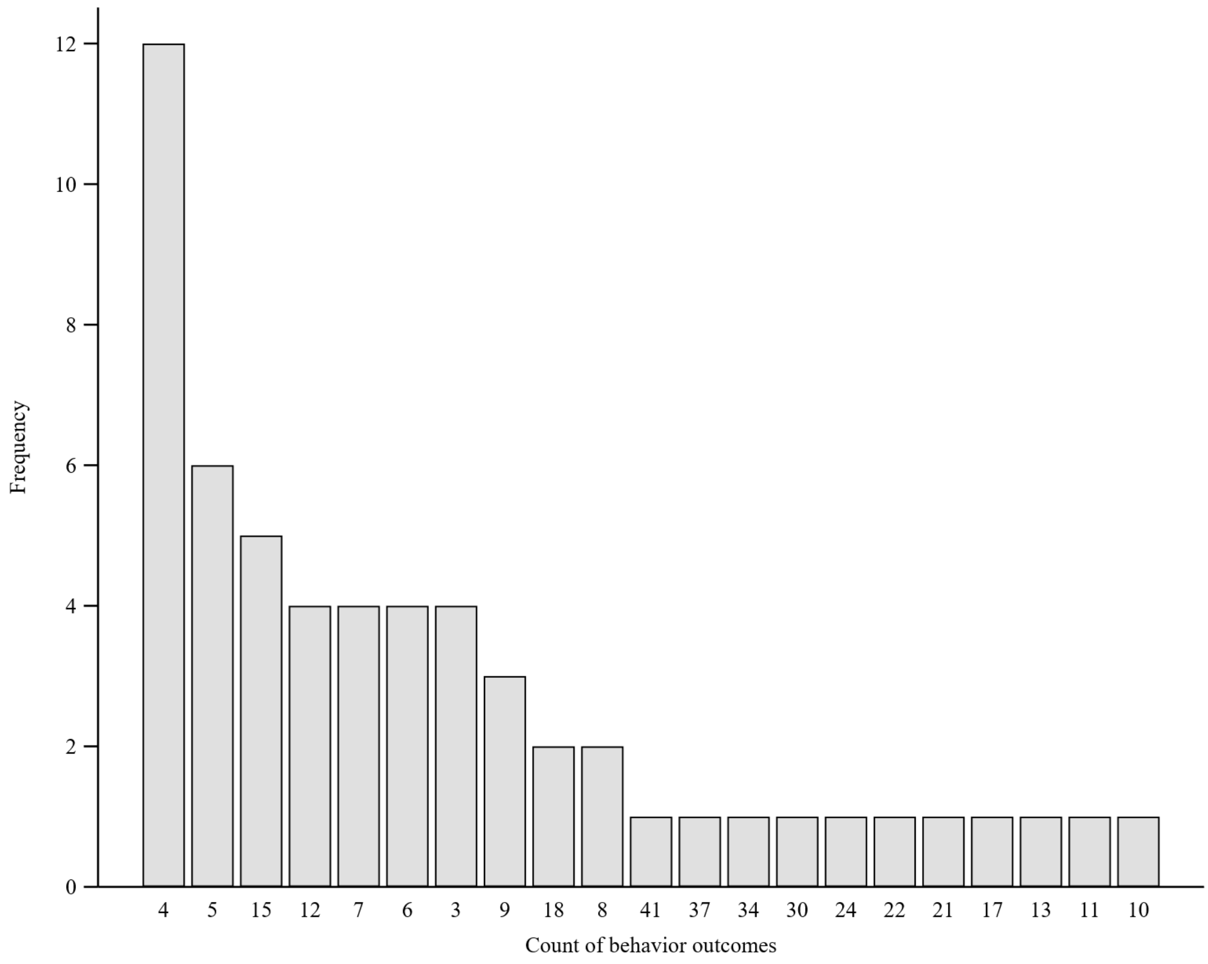

Figure S4. Quantity of outcomes used in clusters procedures.

REFERENCES

1. Androutsos O, Moschonis G, Mavrogianni C, Roma-Giannikou E, Chrousos GP, Kanaka-Gantenbein C, et al. Identification of lifestyle patterns, including sleep deprivation, associated with insulin resistance in children: the Healthy Growth Study. Eur J Clin Nutr. março de 2014;68(3):344–9.

2. Bel-Serrat S, Mouratidou T, Santaliestra-Pasías AM, Iacoviello L, Kourides YA, Marild S, et al. Clustering of multiple lifestyle behaviours and its association to cardiovascular risk factors in children: the IDEFICS study. Eur J Clin Nutr. agosto de 2013;67(8):848–54.

3. Berlin KS, Kamody RC, Thurston IB, Banks GG, Rybak TM, Ferry RJ. Physical Activity, Sedentary Behaviors, and Nutritional Risk Profiles and Relations to Body Mass Index, Obesity, and Overweight in Eighth Grade. Behav Med. 2 de janeiro de 2017;43(1):31–9.

4. Boone-Heinonen J, Gordon-Larsen P, Adair LS. Obesogenic Clusters: Multidimensional Adolescent Obesity-related Behaviors in the U.S. Ann Behav Med. dezembro de 2008;36(3):217–30.

5. Busch V, Van Stel HF, Schrijvers AJ, de Leeuw JR. Clustering of health-related behaviors, health outcomes and demographics in Dutch adolescents: a cross-sectional study. BMC Public Health. dezembro de 2013;13(1):1118.

6. Cameron AJ, Crawford DA, Salmon J, Campbell K, McNaughton SA, Mishra GD, et al. Clustering of Obesity-Related Risk Behaviors in Children and Their Mothers. Ann Epidemiol. fevereiro de 2011;21(2):95–102.

7. Cuenca-García M, Huybrechts I, Ruiz JR, Ortega FB, Ottevaere C, González-Gross M, et al. Clustering of Multiple Lifestyle Behaviors and Health-related Fitness in European Adolescents. J Nutr Educ Behav. novembro de 2013;45(6):549–57.

8. Dumuid D, Olds T, Martín-Fernández J-A, Lewis LK, Cassidy L, Maher C. Academic Performance and Lifestyle Behaviors in Australian School Children: A Cluster Analysis. Health Educ Behav. dezembro de 2017;44(6):918–27.

9. Fernández-Alvira JM, De Bourdeaudhuij I, Singh AS, Vik FN, Manios Y, Kovacs E, et al. Clustering of energy balance-related behaviors and parental education in European children: the ENERGY-project. Int J Behav Nutr Phys Act. 2013;10(1):5.

10. Ferrar K, Golley R. Adolescent Diet and Time Use Clusters and Associations With Overweight and Obesity and Socioeconomic Position. Health Educ Behav. junho de 2015;42(3):361–9.

11. Fleary SA. Combined Patterns of Risk for Problem and Obesogenic Behaviors in Adolescents: A Latent Class Analysis Approach. J Sch Health. março de 2017;87(3):182–93.

12. Gubbels JS, Kremers SP, Goldbohm RA, Stafleu A, Thijs C. Energy balance-related behavioural patterns in 5-year-old children and the longitudinal association with weight status development in early childhood. Public Health Nutr. agosto de 2012;15(8):1402–10.

13. Gubbels JS, Kremers SP, Stafleu A, Goldbohm R, de Vries NK, Thijs C. Clustering of energy balance-related behaviors in 5-year-old children: Lifestyle patterns and their longitudinal association with weight status development in early childhood. Int J Behav Nutr Phys Act. 2012;9(1):77.

14. Hartz J, Yingling L, Ayers C, Adu-Brimpong J, Rivers J, Ahuja C, et al. Clustering of Health Behaviors and Cardiorespiratory Fitness Among U.S. Adolescents. J Adolesc Health. maio de 2018;62(5):583–90.

15. Huh J, Riggs NR, Spruijt-Metz D, Chou C-P, Huang Z, Pentz M. Identifying Patterns of Eating and Physical Activity in Children: A Latent Class Analysis of Obesity Risk. Obesity. março de 2011;19(3):652–8.

16. Iannotti RJ, Wang J. Patterns of Physical Activity, Sedentary Behavior, and Diet in U.S. Adolescents. J Adolesc Health. agosto de 2013;53(2):280–6.

17. Kontogianni MD, Farmaki A-E, Vidra N, Sofrona S, Magkanari F, Yannakoulia M. Associations between Lifestyle Patterns and Body Mass Index in a Sample of Greek Children and Adolescents. J Am Diet Assoc. fevereiro de 2010;110(2):215–21.

18. Landsberg B, Plachta-Danielzik S, Lange D, Johannsen M, Seiberl J, Müller MJ. Clustering of lifestyle factors and association with overweight in adolescents of the Kiel Obesity Prevention Study. Public Health Nutr. outubro de 2010;13(10A):1708–15.

19. Laxer RE, Brownson RC, Dubin JA, Cooke M, Chaurasia A, Leatherdale ST. Clustering of risk-related modifiable behaviours and their association with overweight and obesity among a large sample of youth in the COMPASS study. BMC Public Health [Internet]. dezembro de 2017 [citado 1º de outubro de 2018];17(1). Disponível em: http://bmcpublichealth.biomedcentral.com/articles/10.1186/s12889-017-4034-0

20. Laxer RE, Cooke M, Dubin JA, Brownson RC, Chaurasia A, Leatherdale ST. Behavioural patterns only predict concurrent BMI status and not BMI trajectories in a sample of youth in Ontario, Canada. Fürnsinn C, organizador. PLOS ONE. 2 de janeiro de 2018;13(1):e0190405.

21. Lazzeri G, Panatto D, Domnich A, Arata L, Pammolli A, Simi R, et al. Clustering of health-related behaviors among early and mid-adolescents in Tuscany: results from a representative cross-sectional study. J Public Health. 1º de março de 2018;40(1):e25–33.

22. Leech, McNaughton SA, Timperio A. Clustering of children’s obesity-related behaviours: associations with sociodemographic indicators. Eur J Clin Nutr. maio de 2014;68(5):623–8.

23. Leech RM, McNaughton SA, Timperio A. Clustering of diet, physical activity and sedentary behaviour among Australian children: cross-sectional and longitudinal associations with overweight and obesity. Int J Obes. julho de 2015;39(7):1079–85.

24. Lioret S, Touvier M, Lafay L, Volatier J-L, Maire B. Dietary and Physical Activity Patterns in French Children Are Related to Overweight and Socioeconomic Status. J Nutr. 1º de janeiro de 2008;138(1):101–7.

25. Magee CA, Caputi P, Iverson DC. Patterns of health behaviours predict obesity in Australian children: Health profiles and obesity. J Paediatr Child Health. abril de 2013;49(4):291–6.

26. Mandic S, Bengoechea EG, Coppell KirstenJ, Spence JohnC. Clustering of (Un)Healthy Behaviors in Adolescents from Dunedin, New Zealand. Am J Health Behav. 1º de maio de 2017;41(3):266–75.

27. Marttila-Tornio K, Ruotsalainen H, Miettunen J, Männikkö N, Kääriäinen M. Clusters of health behaviours and their relation to body mass index among adolescents in Northern Finland. Scand J Caring Sci. 30 de outubro de 2019;scs.12769.

28. Miguel-Berges ML, Zachari K, Santaliestra-Pasias AM, Mouratidou T, Androutsos O, Iotova V, et al. Clustering of energy balance-related behaviours and parental education in European preschool children: the ToyBox study. Br J Nutr. 28 de dezembro de 2017;118(12):1089–96.

29. Moschonis G, Mavrogianni C, Karatzi K, Iatridi V, Chrousos GP, Lionis C, et al. Increased physical activity combined with more eating occasions is beneficial against dyslipidemias in children. The Healthy Growth Study. Eur J Nutr. abril de 2013;52(3):1135–44.

30. Moschonis G, Kalliora AC, Costarelli V, Papandreou C, Koutoukidis D, Lionis C, et al. Identification of lifestyle patterns associated with obesity and fat mass in children: the Healthy Growth Study. Public Health Nutr. março de 2014;17(3):614–24.

31. Nuutinen T, Lehto E, Ray C, Roos E, Villberg J, Tynjälä J. Clustering of energy balance-related behaviours, sleep, and overweight among Finnish adolescents. Int J Public Health. novembro de 2017;62(8):929–38.

32. Ottevaere C, Huybrechts I, Benser J, De Bourdeaudhuij I, Cuenca-Garcia M, Dallongeville J, et al. Clustering patterns of physical activity, sedentary and dietary behavior among European adolescents: The HELENA study. BMC Public Health [Internet]. dezembro de 2011 [citado 1º de outubro de 2018];11(1). Disponível em: https://bmcpublichealth.biomedcentral.com/articles/10.1186/1471-2458-11-328

33. Pereira S, Katzmarzyk P, Gomes T, Borges A, Santos D, Souza M, et al. Profiling Physical Activity, Diet, Screen and Sleep Habits in Portuguese Children. Nutrients. 2 de junho de 2015;7(6):4345–62.

34. Pérez-Rodrigo C, Gil Á, González-Gross M, Ortega R, Serra-Majem L, Varela-Moreiras G, et al. Clustering of Dietary Patterns, Lifestyles, and Overweight among Spanish Children and Adolescents in the ANIBES Study. Nutrients. 28 de dezembro de 2015;8(1):11.

35. Platat C, Perrin A-E, Oujaa M, Wagner A, Haan M-C, Schlienger J-L, et al. Diet and physical activity profiles in French preadolescents. 2006;8.

36. Riggs NR, Huh J, Chou C-P, Spruijt-Metz D, Pentz MA. Executive function and latent classes of childhood obesity risk. J Behav Med. dezembro de 2012;35(6):642–50.

37. Rodrigues PRM, Pereira RA, Santana AMS, Gama A, Carvalhal IM, Nogueira H, et al. Patterns of lifestyle-related behaviors and parents' overweight are associated with increased body adiposity in schoolchildren: a cross-sectional study in Portugal. Nutrire. dezembro de 2017;42(1):23.

38. Santaliestra-Pasías AM, Mouratidou T, Reisch L, Pigeot I, Ahrens W, Mårild S, et al. Clustering of lifestyle behaviours and relation to body composition in European children. The IDEFICS study. Eur J Clin Nutr. julho de 2015;69(7):811–6.

39. Seghers J, Rutten C. Clustering of multiple lifestyle behaviours and its relationship with weight status and cardiorespiratory fitness in a sample of Flemish 11- to 12-year-olds. Public Health Nutr. novembro de 2010;13(11):1838–46.

40. Spengler S, Mess F, Mewes N, Mensink GB, Woll A. A cluster-analytic approach towards multidimensional health-related behaviors in adolescents: the MoMo-Study. BMC Public Health. dezembro de 2012;12(1):1128.

41. Spengler S, Mess F, Schmocker E, Woll A. Longitudinal associations of health-related behavior patterns in adolescence with change of weight status and self-rated health over a period of 6 years: results of the MoMo longitudinal study. BMC Pediatr. dezembro de 2014;14(1):242.

42. Turner K, Dwyer JJM, Edwards AM, Allison KR. Clustering of Specific Health-related Behaviours: Among Toronto Adolescents. Can J Diet Pract Res. setembro de 2011;72(3):e155–60.

43. Sluis ME, Lien N, Twisk JW, Steenhuis IH, Bere E, Klepp K-I, et al. Longitudinal associations of energy balance-related behaviours and cross-sectional associations of clusters and body mass index in Norwegian adolescents. Public Health Nutr. outubro de 2010;13(10A):1716–21.

44. Veloso SM, Matos MG, Carvalho M, Diniz JA. Psychosocial Factors of Different Health Behaviour Patterns in Adolescents: Association with Overweight and Weight Control Behaviours. J Obes. 2012;2012:1–10.

45. Yen L-L, Chiu C-J, Wu W-C, Pan L-Y. Aggregation of Health Behaviors among Fourth Graders in Northern Taiwan. J Adolesc Health. setembro de 2006;39(3):435–42.

46. Azeredo CM, Levy RB, Peres MFT, Menezes PR, Araya R. Patterns of health-related behaviours among adolescents: a cross-sectional study based on the National Survey of School Health Brazil 2012. BMJ Open. novembro de 2016;6(11):e011571.

47. Collese TS, De Moraes ACF, Fernández-Alvira JM, Michels N, De Henauw S, Manios Y, et al. How do energy balance-related behaviors cluster in adolescents? Int J Public Health. março de 2019;64(2):195–208.

48. Dantas M, dos Santos M, Lopes L, Guedes D, Guedes M, Oesterreich S. Clustering of Excess Body Weight-Related Behaviors in a Sample of Brazilian Adolescents. Nutrients. 15 de outubro de 2018;10(10):1505.

49. Jureša, V, Petrović D. Behavioral Pattern of Overweight and Obese School Children. Coll Antropol. 2012;36(1):139.

50. Maia EG, Mendes LL, Pimenta AM, Levy RB, Claro RM. Cluster of risk and protective factors for obesity among Brazilian adolescents. Int J Public Health. maio de 2018;63(4):481–90.

51. Matias TS, Silva KS, Silva JA da, Mello GT de, Salmon J. Clustering of diet, physical activity and sedentary behavior among Brazilian adolescents in the

national school - based health survey (PeNSE 2015). BMC Public Health [Internet]. dezembro de 2018 [citado 1º de outubro de 2018];18(1). Disponível em: https://bmcpublichealth.biomedcentral.com/articles/10.1186/s12889-018-6203-1

52. Matias TS, Lopes MVV, de Mello GT, Silva KS. Clustering of obesogenic behaviors and association with body image among Brazilian adolescents in the national school-based health survey (PeNSE 2015). Prev Med Rep. 1º de dezembro de 2019;16:101000.

53. Sena E de MS, Muraro AP, Rodrigues PRM, Fiuza RF de P, Ferreira MG. RISK BEHAVIOUR PATTERNS FOR CHRONIC DISEASES AND ASSOCIATED FACTORS AMONG ADOLESCENTS. Nutr Hosp [Internet]. 28 de julho de 2017 [citado 2 de outubro de 2019];34(4). Disponível em: http://revista.nutricionhospitalaria.net/index.php/nh/article/view/666

54. Dumuid D, Olds T, Lewis LK, Martin-Fernández JA, Barreira T, Broyles S, et al. The adiposity of children is associated with their lifestyle behaviours: a cluster analysis of school-aged children from 12 nations: Children's adiposity relates to their lifestyle behaviours. Pediatr Obes. fevereiro de 2018;13(2):111–9.

55. Dumuid D, Olds T, Lewis LK, Martin-Fernández JA, Katzmarzyk PT, Barreira T, et al. Health-Related Quality of Life and Lifestyle Behavior Clusters in School-Aged Children from 12 Countries. J Pediatr. abril de 2017;183:178-183.e2.

56. Moraes ACF, Fernández-Alvira JM, Rendo-Urteaga T, Julián-Almárcegui C, Beghin L, Kafatos A, et al. Effects of clustering of multiple lifestyle-related behaviors on blood pressure in adolescents from two observational studies. Prev Med. janeiro de 2016;82:111–7.

57. Moreira NF, da Veiga GV, Santaliestra-Pasías AM, Androutsos O, Cuenca-García M, de Oliveira ASD, et al. Clustering of multiple energy balance related behaviors is associated with body fat composition indicators in adolescents: Results from the HELENA and ELANA studies. Appetite. janeiro de 2018;120:505–13.
